# Melt stripping and agglutination of pyroclasts during the explosive eruption of low viscosity magmas

**DOI:** 10.1038/s41467-022-28633-w

**Published:** 2022-02-22

**Authors:** Thomas J. Jones, James K. Russell, Richard J. Brown, Lea Hollendonner

**Affiliations:** 1 Department of Earth, Ocean and Ecological Sciences, University of Liverpool, Liverpool, L69 3GP UK; 2 Department of Earth, Ocean & Atmospheric Sciences, University of British Columbia, Vancouver, BC V6T 1Z4 Canada; 3 Department of Earth Sciences, Durham University, Lower Mountjoy, South Road, Durham, DH1 3LE UK; 4 Department of Physics, University of Regensburg, Universitätsstraße 31, 93053 Regensburg, Germany

**Keywords:** Volcanology, Volcanology, Natural hazards

## Abstract

Volcanism on Earth and on other planets and satellites is dominated by the eruption of low viscosity magmas. During explosive eruption, high melt temperatures and the inherent low viscosity of the fluidal pyroclasts allow for substantial post-fragmentation modification during transport obscuring the record of primary, magmatic fragmentation processes. Here, we show these syn-eruption modifications, in the form of melt stripping and agglutination, to be advantageous for providing fundamental insights into lava fountain and jet dynamics, including eruption velocities, grain size distributions and melt physical properties. We show how enigmatic, complex pyroclasts termed pelletal lapilli form by a two-stage process operating above the magmatic fragmentation surface. Melt stripping from pyroclast surfaces creates a spray of fine melt droplets whilst sustained transport in the fountain allows for agglutination and droplet scavenging, thereby coarsening the grain size distribution. We conclude with a set of universal regime diagrams, applicable for all fluidal fountain products, that link fundamental physical processes to eruption conditions and melt physical properties.

## Abstract (web summary)

The pyroclast properties and features can provide insights into the dynamics of explosive eruptions of low viscosity magma. Here, the authors show how lava droplets, or pyroclasts are subject to melt removal and melt addition during transport in a gas jet and present a method to reconstruct eruption conditions from the pyroclast textures.

## Introduction

The eruption of low viscosity magmas ($\lesssim {10}^{3}$ Pa s) is the most frequent and volumetrically abundant form of volcanism on Earth and on other planets and satellites^1–3^. Explosive eruption of these magmas (i.e., episodes or events that produce pyroclasts) commonly occurs through lava fountaining, jets and discrete explosions (i.e., Hawaiian, Strombolian, and Violent Strombolian eruption styles). These styles of explosive eruption are observed for magmas spanning a wide range of melt compositions including basalt, alkaline mafic melts (e.g., kimberlite, nephelinite), and carbonatite^4–8^. Pyroclasts generated in these systems serve as direct evidence of the mechanisms and intensity of fragmentation processes in explosive eruptions. However, the pyroclasts are generated at temperatures well above their glass transition temperatures (*T*_g_) and for pyroclasts lapilli sized, or greater, cooling commonly occurs on timescales longer than their transport duration^9–12^. One consequence is that they are subject to post-fragmentation processes, which modify pyroclast features, thereby obscuring those generated by the primary magmatic fragmentation event^9,12–16^. These modifications, however, provide fundamental insights into eruption dynamics and melt physical properties.

Previous studies have shown how pyroclast properties and features can provide insights into the dynamics of explosive eruptions of low viscosity magma. For example, the use of fluidal pyroclasts such as Pele’s tears and spheres (i.e., achneliths) have been used to inform on the relative time above the glass transition temperature during which surface tension driven relaxation acts to reshape irregular melt droplets into perfect spheres^9^. The vesicularity of pyroclasts can also provide information on the thermal structure of lava fountains^7,12,15,17,18^. It has been shown that pyroclasts within the inner, thermally insulated, part of the fountain undergo further bubble growth and coalescence^17^. These previous studies^9,11,14,17–21^ have provided coherent semi-quantitative to quantitative physical explanations for the diversity of ultramafic and mafic pyroclasts—except for pelletal lapilli—which despite being found globally in a wide range of volcaniclastic deposits^22–27^ remain controversial in origin^24,28–31^. Texturally, pelletal lapilli comprise a rock or mineral fragment core enclosed by a thin coat of juvenile material^26^. These pyroclasts are typically, <1–60 mm in size^28^, hence the term, lapilli, however, we prefer to use the term pelletal pyroclast to avoid invoking a specific, restricted size range that does not inform on their origin. Previously, they have been referred to by a variety of names such as spinning droplets^23,32^, composite spheroidal lapilli^31^, concentric-shelled lapilli^30^, cannonballs^33^, and cored bombs^34–36^.

The grain size distribution (GSD) of pyroclasts produced by the magmatic fragmentation event and the total grain size distribution (TGSD) of the resulting tephra deposit provide crucial, quantitative insights into the fragmentation style and energy, pyroclast dispersal and atmospheric residence time^37,38^. However, standard field-based techniques used to quantify GSDs at ultramafic and mafic volcanoes are notoriously problematic. The deposits generally have poor preservation and can be rheomorphic in nature^11,20^, are often redistributed by coeval lava flows^39^, fractures can heal during eruption^40^, pyroclasts are susceptible to secondary fragmentaton^2,16,41^ and scoria cones feature a complex stratigraphy, commonly resulting from numerous indistinguishable explosive events or episodes. Currently we rely on, and are limited to, real-time monitoring of active volcanoes via thermal videography to document the GSD produced by explosive mafic eruptions^16,38,42^. Such methods require extensive data processing and targeted camera deployment and, therefore, cannot be used to document eruptions seldom (or never) witnessed (e.g., kimberlite, carbonatite, nephelinite).

Here, we develop a new model for the formation of pelletal pyroclasts using observations on pyroclasts collected from pyroclastic fallout deposits at the Igwisi Hills volcanoes, Tanzania^4^. We further demonstrate the utility of these pelletal textures for unravelling the dynamics attending explosive eruption of low viscosity magmas. Specifically, the spectrum of textures preserved in these pyroclasts record the sequence and dynamics of pyroclast modification (e.g., melt stripping, agglutination) within the conduit and the lava fountain or jet. We show how (total) grain size distributions produced during explosive eruption episodes can be both coarsened and fined during transport, after primary fragmentation. A final result of our analysis is encapsulated in a set of novel and universal regime diagrams for low viscosity magmas (e.g., basalt, kimberlite, carbonatite) that link pyroclast textures (e.g., melt-coated crystals) to both the eruption conditions (e.g., jet velocity) and physical properties of the melt or magma (e.g., surface tension). Additionally, although our focus here is on volcanic melts, our regime diagrams and models are equally applicable to ejecta curtains produced by meteorite impacts^43^, particles ejected during nuclear accidents^44^ and droplet formation/spray coating of industrial fluids having similar viscosity (e.g., pharmaceuticals, paints, plastics, or foods).

## Results

### Field location and observations

**Fig. 1:**
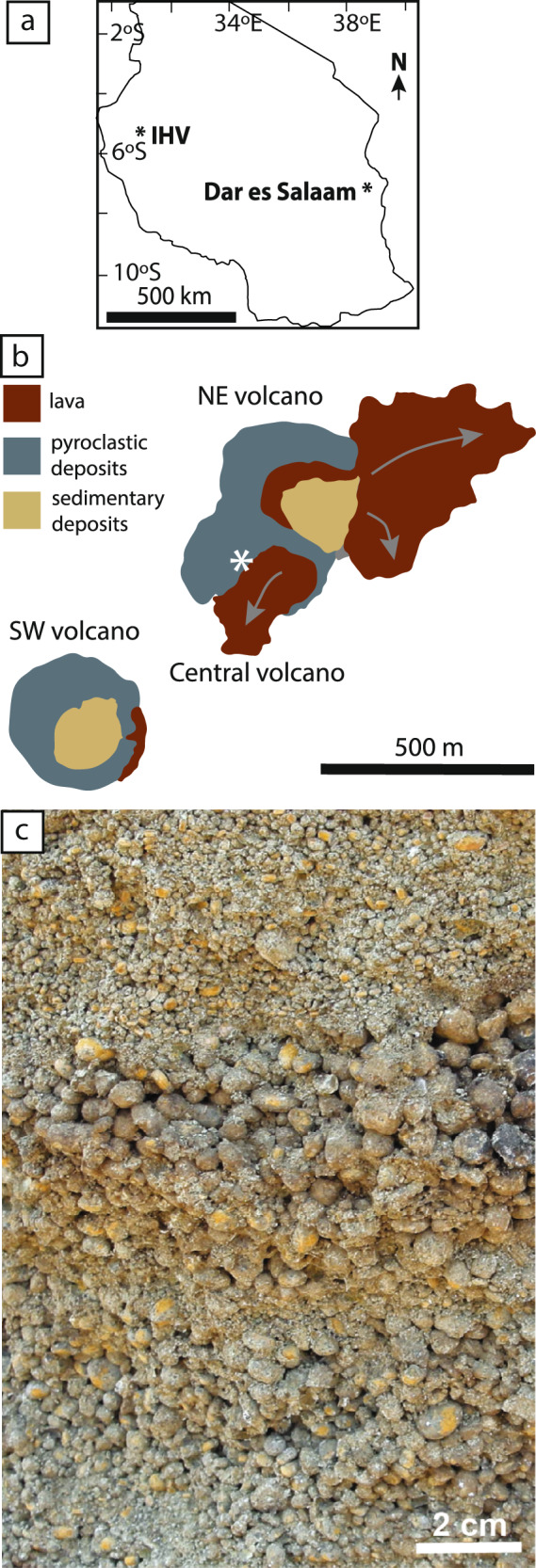
The Igwisi Hills volcanoes (IHV), Tanzania. **a** Outline map of Tanzania with the city Dar es Salaam and the Igwisi Hills volcanoes marked. **b** Geological map of the Igwisi Hills volcanoes with our sample site marked by the asterisk (−4.887884° latitude, 31.933439° longitude). The map is adapted from Shaikh et al.^47^, with permission. **c** Field photograph taken from Brown et al.^4^ of the bedded pyroclastic fallout deposits sampled as part of this study that make up part of the pyroclastic cone on the west of the Central volcano. The deposits are composed of clast-supported pelletal lapilli. For further detailed deposit descriptions the readers are referred to Brown et al.^4^.

The Igwisi Hills volcanoes (IHV) comprise three small, closely-spaced monogenetic kimberlitic volcanoes on the western margin of the Tanzanian craton^4,45–47^ (Fig. 1a, b). The pyroclastic fallout deposits in this study come from a half-cone on the NW sector of the Central volcano^4^, that resulted from early explosive eruptions of lithic-rich pyroclastic material, followed by the eruption of lithic-poor, juvenile-rich pyroclastic material. A late-stage viscous kimberlite lava coulee fills the volcano’s crater. The half-cone comprises alternating beds of (i) clast-supported, very well sorted (*σ*_Φ_ = 0.75–1), 40–70% juvenile clasts that lack cores, and 60–30% subspherical pyroclasts clasts, 0.25–8 mm in diameter, and (ii) clast-supported, well sorted (*σ*_Φ_ = 1.5–2), dense to poorly vesicular angular pyroclasts 0.5–50 mm in diameter, with <15 vol.% of pelletal clasts. The subspherical pyroclasts, found in all beds, consist of an olivine crystal or infrequently a lithic clast enclosed in a concentric shell of quenched melt. Beds are between 3 and 100 cm thick, laterally pinch out and dip outwards between 15–32°. This study focusses on samples from a subspherical pyroclast-rich tephra bed (Fig. 1c).

### Pyroclast textures

**Fig. 2:**
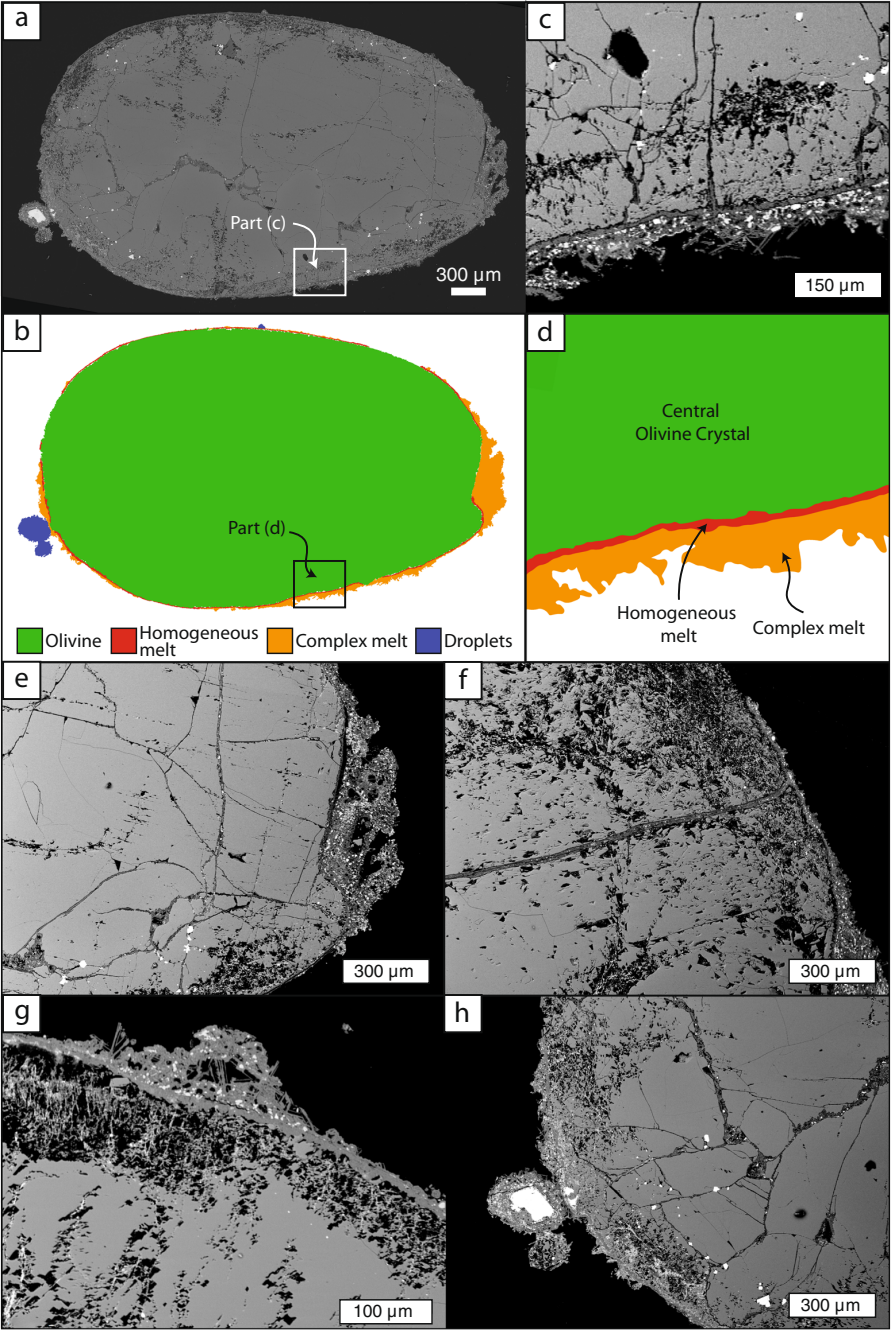
SEM images showcasing the textural features preserved in olivine-cored pelletal pyroclasts. **a** A large image mosaic of an IHV complex pelletal pyroclast with **b** the accompanying trace and labelled features. Green represents the olivine crystal, red the homogenous melt, orange the complex melt and dark blue represents adhering droplets. **c** A close-up view of the olivine crystal—melt interface, where a homogeneous quenched melt layer (now glass) can be seen at the interface overlain in places by more crystal-rich material. The accompanying trace with feature identifications are shown in **d** at the same scale. **e** Preferential thickening of the coating within a depression on the olivine crystal surface. **f** Internal melt-filled cracks that connect to the surface and the melt coating. **g** A spreading droplet on top of the quenched, homogeneous layer. **h** Sticking, circular, undeformed droplets tacked on to the main pyroclast.

In this study, 30 pyroclasts were imaged under a scanning electron microscope (SEM). Their short axis diameters ranged in size from 0.9 to 7.0 mm. A complete set of SEM imagery and pyroclast traces can be found in the online supplementary information as Fig. S1. Of these 30 pyroclasts, 29 are pelletal pyroclasts featuring an ellipsoidal olivine core (Fig. 2) and one is an olivine crystal completely devoid of any adhering material. Most pelletal pyroclasts have two coatings that can be continuous or discontinuous. The inner coating is a layer of homogeneous (crystal and vesicle free) glass that coats the olivine crystal surface. This homogeneous glass mantles the olivine crystals, infilling irregularities on the exterior olivine surfaces. The outer coating comprises a discontinuous or continuous coating of crystal-rich glass that may also contain vesicles (Fig. 2a–d). In some cases this pore space (i.e., vesicles) may be enhanced by secondary alteration that is commonly observed in kimberlite deposits^48^. This outer, complex glass coating contains approximately 25% crystals and 12% bubbles by modal area abundance (see “Methods” section). There is a sharp contact between the homogenous and complex glasses. No preferred crystal orientations are observed within the complex glass. However, vesicles close to the homogeneous, complex melt interface are sometimes elongated, with their long axis orientated parallel to the interface contact. One pelletal lapilli sample appears not to have the inner coating of homogeneous glass but, instead, is coated only by the complex crystal-rich, vesicular glass. Energy-dispersive X-ray spectroscopy (EDS) analysis releveled that the homogeneous and complex glasses are chemically indistinguishable (Fig. S2).

The olivine crystals are ellipsoidal and have numerous internal cross-cutting cracks that commonly contain mineral inclusions and quenched melt. These textural observations are consistent with previous work conducted on lava-hosted olivine crystals from IHV^49,50^. Two pyroclasts are broken, one has a resulting clean, uncoated surface. The outer crystal-rich coating varies in thickness and is often thicker in embayments on the olivine crystal surface, suggesting that the pyroclasts are, at least in part, aerodynamically shaped (Fig. 2e). Some internal cracks intersect the exterior surface of the olivine grain and have been infiltrated by melt (now quenched; Fig. 2f). Some pyroclasts have vesicular, crystal-rich droplets (i.e., texturally the same as the outer coating) attached to their exterior surfaces. Only droplets that can be clearly identified, based on their texture, from bulges in the complex coating are identified and referred to separately. The droplets range in shape from circular to semi-spheres with higher aspect ratios (Fig. 2g, h).

### Image analysis results

**Fig. 3:**
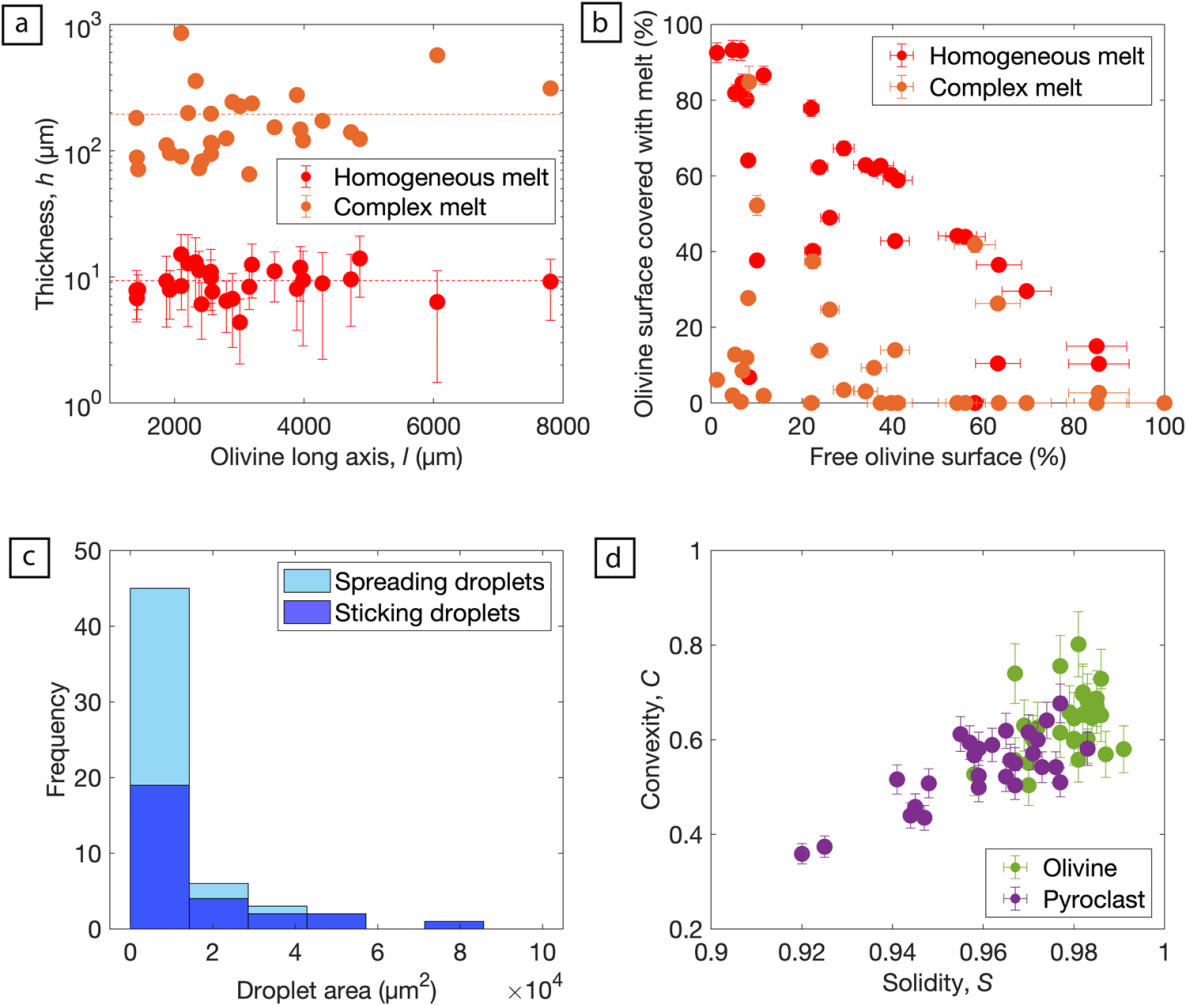
Quantitative image analysis results. **a** The mean thickness of the homogeneous, quenched melt layer (red) and the maximum thickness of the complex, crystal rich, vesicular melt coating (orange) as a function of olivine long axis length. Error bars represent the thickness variability, unique to each individual pyroclast. **b** The amount of free olivine crystal surface plotted against the proportion (%) of the olivine crystal surface covered by homogeneous melt and complex melt. Error bars represent one standard deviation obtained by repeat measurements on a representative pyroclast. **c** A frequency histogram (*n* = 57) of the melt droplet area (spreading and sticking) for all pyroclasts analysed in this study. **d** Convexity, *C* and solidity, *S* shape factors for the central olivine crystal and the entire pyroclast (olivine crystal and coating). Error bars represent one standard deviation obtained by repeat measurements on a representative pyroclast. All accompanying data can be found in the online Supplementary Data 1.

We have quantified the textural properties of these pyroclasts via image analysis (see “Methods” section). The melt coatings can be grouped into two types: (1) a homogeneous layer that, where present, is of uniform thickness measuring ~10 µm; (2) a complex crystal-rich, vesicular layer that varies in thickness with maximum thicknesses averaging ~195 µm. Over the pyroclast size range investigated, melt thicknesses or surface coverage do not vary as a function of olivine grain size (Fig. 3a). The average coating covers 67% of the olivine surface and for most pyroclasts the homogeneous layer dominates the contact with the crystal (Fig. 3b). We also measured the area of texturally distinct droplets that adhere to the pyroclast (Fig. 3c). These form part of the melt droplet size-distribution within the gas-pyroclast mixture that could be scavenged by the larger, pelletal pyroclasts. Texturally these droplets can be further split into undeformed, spherical droplets that stick to the surface, termed sticking droplets (Fig. 2h) and deformed, spreading droplets (Fig. 2g). These have an average area of 13,436 and 6262 µm^2^, respectively and, assuming an original circular shape, droplet radii of 65 and 45 µm, respectively (Fig. 3c). The smallest droplet identified had an area of 36 µm^2^ (~3 µm radius). Lastly, to document the pyroclast shape, the convexity (*C* = *P*_p_/*P*_H_) and solidity (*S* = *A*_H_/*A*_P_) shape factors were calculated, where *P*_p_ is pyroclast perimeter, *P*_H_ is perimeter of the bounding convex hull, *A*_P_ is pyroclast area and *A*_H_ is area of the bounding convex hull. *C* describes the small-scale concavities on the particle surface, referred to as textural roughness and *S* describes the roughness and irregularities on a particle scale^51^. Both the central olivine crystals and pyroclasts (crystal and all quenched melt) have a narrow range of solidity values, with average values of 0.98 and 0.95, respectively (Fig. 3d). The convexity values are much lower for the pyroclasts (0.53 on average) relative to the olivine crystals (0.64 on average) reflecting the increased textural roughness provided by adherence of melt coats and droplets. All image analysis results can be found in Supplementary Data 1.

## Discussion

**Fig. 4:**
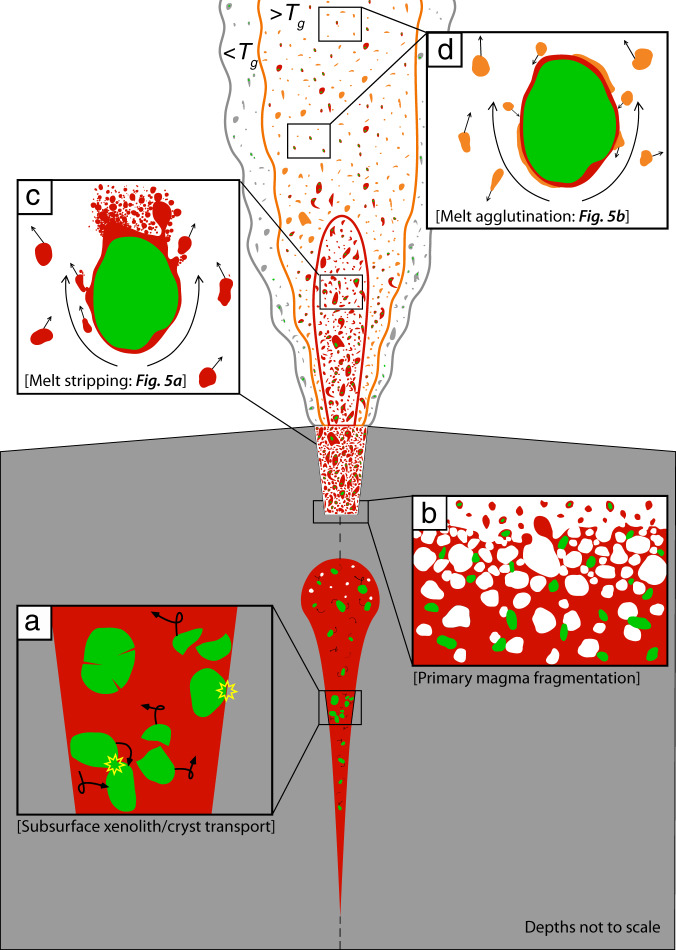
The key dynamic processes that lead to the pyroclast textures observed. The colour scheme is the same as previous figures where, green represents olivine crystals, red represents the homogenous melt and orange represents the complex melt. Gas is shown in white, and the grey at the fountain/jet edge indicates the cooler portion of the fountain/jet that is below the glass transition temperature, *T*_g_. **a** Within conduit/dyke mechanical abrasion of olivine crystal cargo. **b** A simple depiction of the primary magma disruption and formation of melt-coated crystals and melt droplets at the free surface. We note that these primary fragmentation processes are likely to be highly variable and complex. **c** Stripping of the mobile, high-temperature melt from crystal surfaces (see Fig. 5a) and **d** in the lower velocity part of the fountain, in-flight particle agglutination (see Fig. 5b). Note that agglutination processes shown in **d** can also occur in the subsurface when velocities are reduced due to conduit widening, for example.

The pyroclast textures described here, from the Igwisi Hills volcanoes, record a series of ascent and eruption processes (Fig. 4). These processes can be ordered by depth within the volcanic system. During vigorous, rapid (>4 m s^−1^), flow attending subsurface transport, mantle-derived olivine crystals (and other minerals) are reshaped by chemical processes and are abraded by particle–particle interactions to form rounded, ellipsoidal shapes^49,50,52–55^ (Fig. 4a). Less commonly, xenocrysts are evidently fragments of once larger crystals. We interpret these to form by either higher energy (i.e., velocity) collisions^54^ or by the expansion of melt inclusions^56^, potentially within intracrystalline cracks. As the cargo-laden, volatile-rich magma continues to accelerate and reaches the near-surface environment, primary magmatic fragmentation occurs producing a distribution of droplets at the fragmentation surface^2,57,58^ (Fig. 4b). When there is a high proportion of mantle-derived olivine grains, as at IHV and in most kimberlites, many magma droplets will be cored by olivine.

At, and directly above, the fragmentation surface, the olivine-cored, melt-coated pyroclasts are transported in an expanding gas-pyroclast mixture. Differential velocities at the gas-melt interface cause shear. This configuration is naturally unstable and leads to Kelvin–Helmholtz (KH) instabilities, creating undulations at the gas-melt interface^59–61^ that can be further exploited by Rayleigh–Taylor (RT) instabilities. This subsequent development of RT instabilities can lead to melt removal from the pyroclast via ligament formation, break up and droplet production^2,6,59–61^ (Fig. 4c). Collectively, we refer to these processes as melt stripping.

**Fig. 5:**
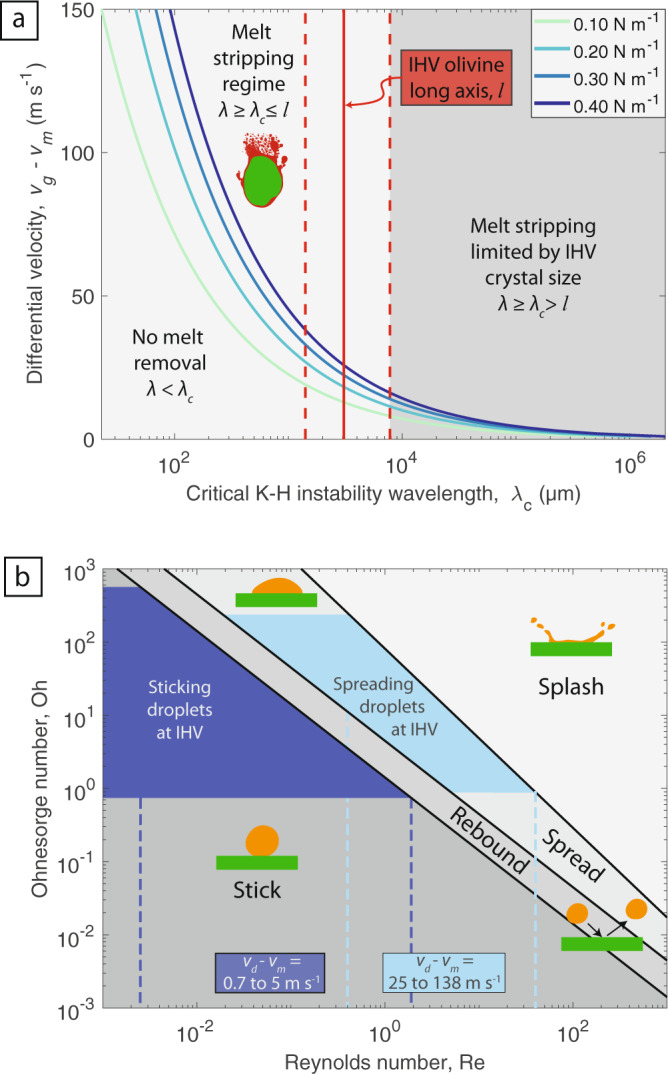
Regime diagrams for melt removal and addition during the eruption of low viscosity magmas. **a** The differential velocity between the gas and homogeneous melt layer against the critical Kelvin–Helmholtz instability wavelength. The four curves show results for four different surface tensions reasonable for melts spanning the ultramafic to mafic compositional spectrum^6, 62, 63^. The dashed red lines mark the observed range in olivine crystal long axis values and the solid red line denotes the mean. **b** Droplet impact regime diagram, relating the impact conditions (Re) to the physical properties of the droplets (Oh). The two shaded regions in dark and light blue correspond to the observed droplet size distribution of both the sticking and spreading droplets respectively (cf. Fig. 3c).

The first part of the melt stripping process, the shear-induced KH instabilities, is therefore a critical step for droplet production via melt stripping from the surface of pyroclasts, which in this case are olivine-cored. Two limiting cases of the KH instability can be described, each controlled by characteristic wavelengths^61^. The first is when the boundary, or vorticity layer is thin, and defines the Kelvin–Helmholtz limit: ${\scriptstyle\sqrt{{\rho }_{{{{{{\mathrm{g}}}}}}}/{\rho }_{{{{{{\mathrm{m}}}}}}}}}\ll 1$. The second is when the vorticity layer is thick and forms the Rayleigh limit at ${\scriptstyle\sqrt{{\rho }_{{{{{{\mathrm{g}}}}}}}/{\rho }_{{{{{{\mathrm{m}}}}}}}}}\gg 1$. For all reasonable estimates of gas and melt density, *ρ*_g_ and *ρ*_m_ respectively, we find that the thin vorticity layer limit is applicable. Under a spatially uniform perturbation, in a planar geometry (i.e., a flat, 2-D plane), surface tension acts to stabilise the instability for all wavenumbers, *k* greater than the critical wavenumber, *k*_c_:

1 $$k \; > \; {k}_{{{{{{\mathrm{c}}}}}}}=\frac{{\rho }_{{{{{{\mathrm{m}}}}}}}{\rho }_{{{{{{\mathrm{g}}}}}}}}{{\rho }_{{{{{{\mathrm{m}}}}}}}+{\rho }_{{{{{{\mathrm{g}}}}}}}}\frac{{({v}_{{{{{{\mathrm{g}}}}}}}-{v}_{{{{{{\mathrm{m}}}}}}})}^{2}}{{\sigma }_{{{{{{{\mathrm{mg}}}}}}}}},$$

where *v*_g_ is gas velocity, *v*_m_ is melt (i.e., pyroclast) velocity, and *σ*_mg_ the surface tension between the melt and gas. Wavenumbers are related to the wavelength by: *λ* = 2*π*/*k*. This relationship is shown in Fig. 5a for *ρ*_m_ = 2670 kg m^−3^, *ρ*_g_ = 1.225 kg m^−3^ and contoured for a range of surface tensions reasonable for melts spanning the ultramafic to mafic compositional spectrum^6,62,63^. Larger differential velocities between the melt layer and the gas will lead to shorter critical instability wavelengths, *λ*_c_. We can relate *λ*_c_ to the long axis of the olivine crystal (*l*) to form a criterion. The length scale, *l* is used, rather than the pyroclast perimeter, because it is the longest dimension in which the shear can act in a uniform direction. Thus, for KH instabilities to form in the melt layer, and potentially lead to melt removal, *λ*_c_ < *l*. Using this criterion in conjunction with our suite of IHV pelletal pyroclasts reveals minimum differential velocities, between the gas and the melt of the pyroclast surface, of 13 to 26 m s^−1^ for the mean *l* (solid red line; Fig. 5a) and for the range of surface tensions adopted. These differential velocities are readily achievable at IHV and for other mafic systems. For example, during the onset of a kimberlite eruption gas velocities are expected^29^ to reach velocities ≫ 600 m s^−1^ and during basaltic lava fountaining gas-melt differential velocities have been modelled^64^ to be ~3 m s^−1^ and ~200 m s^−1^ at Kīlauea and Etna, respectively. Furthermore, rapid clast rotation^23,33^ may also support higher differential velocities.

Following the formation of KH instabilities, a complex array of instabilities can form including transverse destabilisations that lead to ligament formation, extension, and droplet production. By analogy to the droplet breakup mechanisms in an airstream^61,65^, the balance between the aerodynamic pressure (*ρ*_g_[*v*_g_ – *v*_m_]^2^) and the capillary pressure (*σ*_mg_/*d*) sets the size for the droplets produced, *d*. Our image analysis demonstrated that, where present, the homogeneous melt layer coating the crystal surface was of uniform thickness (*h* = 10 µm) irrespective of pyroclast size (Fig. 3a). Given the universality of this thickness, we suggest that for the majority of pelletal pyroclasts the melt stripping process went to equilibrium (i.e., a balance between the aerodynamic and capillary pressure was reached) and that *d* > *h*, making *h* the limiting droplet size that could not be removed from the pyroclast. However, for the case of complete melt removal from a crystal surface at higher differential gas velocities the work of adhesion^66^, incorporating the melt-crystal surface tension, needs to be considered. This parameter is understudied and remains a target for future experimental determination.

The homogeneous melt layer therefore represents the material that could not be removed by the gas shear at the particle interface. We suggest that this layer is mostly homogeneous, crystal and vesicle free simply because its 10 µm thickness is smaller than individual bubbles and crystals. Texturally the melt stripping process has been clear to identify on the olivine-cored pyroclasts found at IHV. However, the xenocryst core is not a prerequisite and the melt stripping processes could equally occur on purely juvenile pyroclasts, such as Pele’s tears or impact ejecta, for example. Without a crystal or lithic core, it is unlikely that any textural record of melt stripping will be preserved—the pyroclast will simply be reduced in volume.

Vesiculated, crystal-bearing and crystal-free melt droplets are generated at the fragmentation surface by a combination of primary magma disruption (Fig. 4b) and by secondary melt stripping at the pyroclast-gas interface (Figs. 4c and 5a). These are then further transported within the conduit and/or in the lava jet at temperatures above the glass transition temperature, *T*_g_. Given the large temperature difference, (Δ*T*) between *T*_g_ and the eruption temperature, *T*_e_ for mafic and ultramafic magmas^67^, the opportunity for syn-transport modification of the pyroclasts is high. Increased conduit diameters, larger distances above the vent and decreased pressures or volumes of liberated gasses all act to reduce differential velocities between the pyroclasts and the gas stream. Differential velocities will ultimately reduce to a point, where melt stripping from the crystal surface is not feasible (Fig. 5a). However, we note that the eruptive jet could feature a heterogenous velocity distribution and pyroclasts could return to the melt stripping regime, potentially multiple times, if local gas-particle velocities once again exceed the threshold. Below the differential gas-particle velocities required for melt stripping, collisions between suspended vesicular melt droplets and (partially) wetted olivine crystals result in late-stage adhesion of melt droplets. We envisage this process to be mostly continual, scavenging melt droplets from the pyroclast mixture and coarsening the eruption’s grain size distribution until pyroclasts reach their *T*_g_. This two-component process (melt removal and agglutination) explains the diverse array of textures observed and ultimately the origin of the pelletal lapilli (Fig. 4d). Agglutination textures have also been observed in the neighbouring NE volcano deposits at IHV^46^, however they are smaller, ash-sized and are interpreted to form entirely within the eruption plume.

When melt droplets impact the homogeneous melt film adhering to the crystal surface (or the crystal surface itself, in the case of complete melt removal) four outcomes are possible^68–71^: (i) the droplet rebounds, (ii) the droplet sticks and retains a spherical shape (Fig. 2h), (iii) the droplet starts to spread (Fig. 2g), and (iv) the droplet splashes. We note that in the textures quenched on natural products outcomes (iii) and (iv) are difficult to differentiate. These four different outcomes occur at a set of distinct conditions. Dimensional analysis reveals that the transition between these impact regimes depends on two key dimensionless groups^68,69^.

First, the Weber number—a ratio of inertial to surface tension forces:

2 $${{{{{\rm{We}}}}}}=\frac{{({v}_{{{{{{\mathrm{d}}}}}}}-{v}_{{{{{{\mathrm{m}}}}}}})}^{2}{\rho }_{{{{{{\mathrm{d}}}}}}}d}{{\sigma }_{{{{{{{\mathrm{mg}}}}}}}}},$$

where *v*_m_ is the velocity of the pyroclast the droplet is impacting, *v*_d_ is the velocity of the droplet impacting the pyroclast, *ρ*_d_ is the droplet density (in most cases ${\rho }_{{{{{{\mathrm{d}}}}}}}\approx {\rho }_{{{{{{\mathrm{m}}}}}}}$), and *d* is the droplet diameter. Note that the differential velocity is different than that expressed in Eq. (1), and here, is the difference in velocity between two discrete particles.

Second, the Laplace number—a ratio of surface tension to viscous forces:

3 $${{{{{\rm{La}}}}}}=\frac{{\rho }_{{{{{{\mathrm{d}}}}}}}{\sigma }_{{{{{{{\mathrm{mg}}}}}}}}d}{{\eta }^{2}},$$

where *η* is the viscosity of the droplet. For droplet impact on wetted surfaces Bai et al.^71^ found that the stick-rebound transition occurs at We ~2, rebound-spread at We ~20 and spread-splash at We ~1320 La^−0.183^.

Furthermore, We and La can be recast to form two additional dimensionless groups, the Reynolds number (Re = We^0.5^/Oh) and the Ohnesorge number (${{{{{\rm{Oh}}}}}}={{{{{{\rm{We}}}}}}}^{0.5}/{{{{{\rm{Re}}}}}}=1/\sqrt{{{{{{\rm{La}}}}}}}$):

4 $${{{{{\mathrm{Re}}}}}}=\frac{({v}_{{{{{{\mathrm{d}}}}}}}-{v}_{{{{{{\mathrm{m}}}}}}}){\rho}_{{{{{{\mathrm{d}}}}}}}d}{\eta},$$

5 $${{{{{\rm{Oh}}}}}}=\frac{\eta }{\sqrt{{\sigma }_{{{{{{{\mathrm{mg}}}}}}}}{\rho }_{{{{{{\mathrm{d}}}}}}}d}}.$$

It is useful to re-define the dimensionless groups in this way because it facilitates comparison to other studies^68^. Re contains the extrinsic properties and Oh, the intrinsic properties of the system. The regimes are shown graphically in Fig. 5b. As an example, assuming reasonable properties for the complex melt (*η* = 0.3 to 34 Pa s, *σ*_mg_ = 0.2 N m^−1^, *ρ*_d_ = 2670 kg m^−3^; see “Methods” section) and for the observed droplet size range, the Oh space for the sticking and the spreading droplets observed at IHV are shown on Fig. 5b by the dark and light blue shaded regions, respectively. Furthermore, through manipulation of Re (Eq. (4)), our observed range of droplet sizes at IHV correspond to maximum differential velocities, between the impacting droplet and olivine cored pyroclast (*v*_d_ – *v*_m_) of 0.7–5 and 21–138 m s^−1^ for the sticking and spreading droplets, respectively. The Bai et al.^71^ experiments, upon which this analysis is based, used wetted surfaces with thicknesses on the order of the droplet diameter. We note that in the natural case the ratio of surface thickness to droplet diameter will be highly variable. In general, thicker wetted surfaces dissipate more energy during impact^72^ and therefore require a higher differential velocity (*v*_d_ – *v*_m_) to cause a transition to the next regime.

Once pyroclasts have cooled to *T*_g_, or if extensive syn-eruptive crystallization increases the suspension viscosity, these processes of melt removal or melt adherence cease to operate. Upon exit from the vent we estimate cooling to *T*_g_ to occur on the order of seconds^9,46^, thus the majority of post magmatic fragmentation modification occurs in the subterranean environment or at ultra-proximal vent regions within the thermally insulated interior of the lava fountain. It is possible that pyroclasts could be further modified by mechanical attrition processes below *T*_g_^53,73,74^, where they remain entrained or become re-entrained in the volcanic plume or within pyroclastic density currents. The abrasive removal of adhering glass/melt coatings on crystals has been documented during other studies of pyroclastic deposits and has been linked to transport distance^75,76^.

Our detailed analysis of pelletal pyroclasts textures has revealed several mostly subterranean, syn-transport processes operating at *T* > *T*_g_ that can modify the textures, shapes, and size-distributions of pyroclasts produced in explosive eruptions of low viscosity magma. We contend that melt stripping is ubiquitous in lava fountains, jets, and explosions, where gas-pyroclast differential velocities are sufficiently high (see Fig. 5a). At these high gas-melt differential velocities melt can be stripped from pyroclast surfaces by fluid dynamic instabilities, releasing fine droplets into the fountain or jet. These critical differential velocities are readily achievable during the eruption of kimberlite, carbonatite, and basalt and during other non-volcanic processes such as meteorite impacts^43^ and nuclear accidents^44^, where a high velocity jet containing molten droplets is produced. Pyroclasts lacking a solid core (e.g., lithic fragment, phenocryst, and xenocryst) are unlikely to provide a textural record of melt stripping processes—the only evidence would be a fining of the pyroclast size distribution. Furthermore, higher viscosity melts will take longer to strip, this reduces the opportunity for the stripping process to complete before the pyroclast cools to the glass transition temperature and becomes immobile. For these reasons melt stripping textures are not readily observed in basaltic pyroclasts—their xenolith or phenocryst content is typically low and melt viscosities can be higher. However, provided differential velocities are sufficiently high (Fig. 5a), basaltic pyroclasts are subject to melt stripping and re-examination of fallout deposits would be appropriate.

**Fig. 6:**
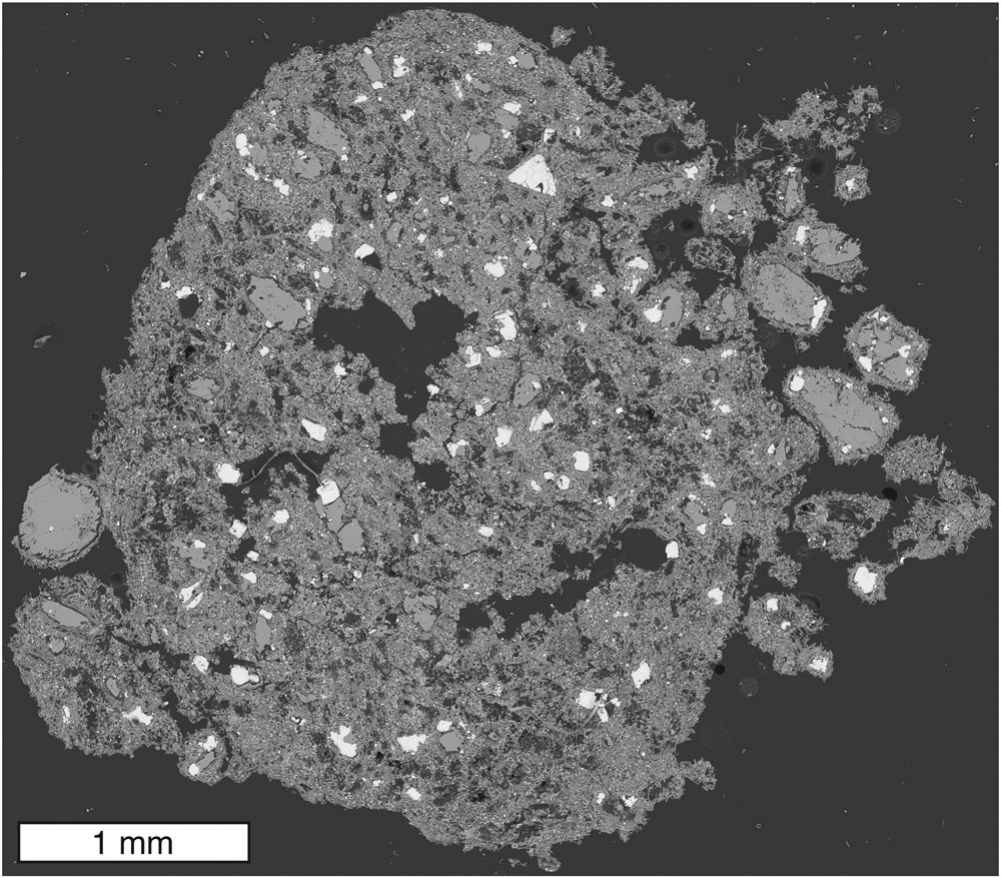
Backscatter scanning electron microscopy image of a juvenile pyroclast (sometimes referred to as a magmaclast in the kimberlite literature^85^). This pyroclast was recovered from the same fallout deposit as shown in Fig. 1. Multiple droplets are observed adhering to the pyroclast, indicating that the processes of agglutination are widespread and not unique to the olivine cored pyroclasts.

Lower differential velocities can be caused by conduit widening, a reduction in mass eruption rate, or be found at locations more distal to vents, for example. At these lower differential velocities, syn-transport agglutination of droplets and pyroclasts is supported, thereby coarsening the pyroclast size distribution. Furthermore, droplet agglutination produces more irregular shapes leading to lower terminal settling velocities, enhancing atmospheric residence and dispersal. These agglutination textures can be identified in all pyroclasts, irrespective of the presence of a solid (olivine) core. Figure 6 shows a juvenile pyroclast from the same fallout deposit at IHV; multiple droplets are observed sticking to the surface of the pyroclast, indicating that agglutination processes are widespread and not unique to the olivine cored pyroclasts. Furthermore, the agglutination of droplets onto surfaces has been observed on pyroclasts and crystals from the IHV NE volcano^46^, on nephelinitic achneliths^77^, lunar impact ejecta^78^, and clasts produced during nuclear accidents^79^ and nuclear weapons tests^80^. The regime diagram (Fig. 5b) is relevant to these scenarios and can be used to uncover the differential velocities that generated the agglutination textures.

These insights into syn-eruptive melt stripping and agglutination complement the growing evidence for a range of TGSD-changing processes^2,16,28,40,41^ that operate after primary magmatic fragmentation during the explosive eruption of low viscosity magmas. Future deposit studies should look for, and consider, these textures which document modification of the original grain size distribution produced by the primary magmatic fragmentation event.

## Methods

### Scanning election microscopy

Thirty individual pyroclasts were carefully extracted from a large ~30 cm by 30 cm by 20 cm tephra sample. Only pyroclasts that could be removed easily and without damage from the lapilli tuff were used in this study. These grains were then sorted into similar sized groups and impregnated with epoxy to form a series of coherent cylinders with a diameter of ~2.5 cm. During impregnation about half of the groups were orientated vertically and half horizontally relative to the long axis of the pyroclast. The cylinders were cut into polished thin sections, centered at the middle of the pyroclasts. The thin sections were then carbon coated using a sputter coater and analyzed under a Philips XL30 SEM in scanning electron mode using with a 15 kV accelerating voltage, a 35 μA beam current, and an average working distance of 12 mm. A series of overlapping images were taken to document the pyroclast, especially its rim. On the same samples, Energy Dispersive X-Ray Spectroscopy (EDS) was performed on a Zeiss Gemini SEM 450 in the SEM Shared Research Facility at the University of Liverpool.

### Image analysis

For each pyroclast the series of overlapping SEM images were manually stitched together in the graphics software, Inkscape. The scale bar length was recorded and used to convert all pixel-based measurements to microns. Then, the olivine crystal, the homogeneous quenched layer, the complex vesicular melt and any droplets were manually traced, each in a new graphics layer and using a different colour. The contact line between both the olivine crystal and the homogeneous melt layer and between the olivine crystal and the complex melt were also manually traced. All of these manual traces were then exported from Inkscape as portable network graphics (png) files and loaded into ImageJ for analysis. The entire pyroclasts were measured for area, perimeter, maximum diameter, minimum diameter, convexity, and solidity. The olivine crystals were measured for area, perimeter, maximum and minimum diameter, convexity, and solidity. The homogeneous melt and complex melt layers had their area measured and their thickness measured at least one hundred and ten times, respectively. The thickness of the complex melt was highly variable so in this study we measured the location where the coating was thickest. The droplets were measured for their area and the contact lines were quantified in terms of their length. To assess the error associated with both manual tracing and the image analysis methods, a pyroclast was separately traced and measured three times. Unless otherwise stated the standard deviation of these three measurements are shown as error bars on the figures.

### Modelling pyroclast agglutination

Our modelling of pyroclast agglutination relies on knowing the magma physical properties, namely viscosity, density, and surface tension. There are few, if any, direct measurements of these physical properties for kimberlite magmas. The melt-gas surface tension values for a wide spectrum of natural silicate melts have a very small range, relative to other magma physical properties. Previous kimberlite studies^6^ have used surface tensions ranging between pure water (0.08 N m^−1^), carbonatite (0.2 N m^−1^) and basalt. The surface tensions of basaltic melts are reasonably well constrained with values ranging from ~0.2 to 0.4 N m^−1^ commonly used^62,63^. For our agglutination calculations we take 0.2 N m^−1^ to be the most suitable value.

Manual tracing was performed on three separate areas of complex melt, on three separate pyroclasts, to determine the modal abundance of bubbles and crystals. Over a total measured area of 118,004 µm^2^, bubbles occupied 13,084 µm^2^ (11%) and crystals occupied 29,282 µm^2^ (25%). We asserted a bubble free, crystal bearing kimberlite melt to have a density^6,81^ of ~3000 kg m^−3^. Using the relationship, *ρ* = (1 – *ϕ*_b_)*ρ*_0_ we calculate the density, *ρ* of the bubble and crystal bearing complex melt to be 2670 kg m^−3^. Where *ϕ*_b_ is the volume fraction of bubbles and *ρ*_0_ is the density of melt and crystals.

Pure kimberlite melts devoid of any crystals and bubbles are expected^6,82,83^ to have viscosities, *μ* between 0.1 and 10 Pa s. The addition of crystals increases the bulk suspension viscosity, *η* by the following relationsip:^84^ *η* = *μ*(1–*ϕ*_p_/*ϕ*_m_)^−2^ where *ϕ*_p_ is the crystal volume fraction and *ϕ*_m_ is the maximum packing fraction. Using the expected range in pure melt viscosities, *ϕ*_p_ = 0.25 and *ϕ*_m_ = 0.55 we suggest that *η*, the complex crystal bearing melt viscosity is within the range 0.3–34 Pa s. The viscosity of three phase (melt, bubbles, crystals) systems is not known for high shear-rates, like those expected within the pyroclastic jet.

## Supplementary information

Supplementary Information

Description of Additional Supplementary Files

Supplementary Data 1

## Data Availability

The data generated or analysed in this study are provided in the Supplementary Information.

## References

[1] Arzilli F (2019). Magma fragmentation in highly explosive basaltic eruptions induced by rapid crystallization. Nat. Geosci..

[2] Jones TJ, Reynolds CD, Boothroyd SC (2019). Fluid dynamic induced break-up during volcanic eruptions. Nat. Commun..

[3] Valentine GA, Gregg TKP (2008). Continental basaltic volcanoes—processes and problems. J. Volcanol. Geotherm. Res..

[4] Brown RJ (2012). Eruption of kimberlite magmas: physical volcanology, geomorphology and age of the youngest kimberlitic volcanoes known on earth (the Upper Pleistocene/Holocene Igwisi Hills volcanoes, Tanzania). Bull. Volcanol..

[5] Brown RJ, Valentine GA (2013). Physical characteristics of kimberlite and basaltic intraplate volcanism and implications of a biased kimberlite record. GSA Bull..

[6] Moss S, Russell JK (2011). Fragmentation in kimberlite: products and intensity of explosive eruption. Bull. Volcanol..

[7] Taddeucci, J., Edmonds, M., Houghton, B., James, M. R. & Vergniolle, S. *The Encyclopedia of Volcanoes* 2nd edn 485–503 (Elsevier, 2015).

[8] Barker DS, Nixon PH (1989). High-Ca, low-alkali carbonatite volcanism at Fort Portal, Uganda. Contrib. Mineral. Petrol..

[9] Porritt LA, Russell JK, Quane SL (2012). Pele’s tears and spheres: examples from Kilauea Iki. Earth Planet. Sci. Lett..

[10] Rader E, Geist D (2015). Eruption conditions of spatter deposits. J. Volcanol. Geotherm. Res..

[11] Sumner JM, Blake S, Matela RJ, Wolff JA (2005). Spatter. J. Volcanol. Geotherm. Res..

[12] Stovall WK, Houghton BF, Hammer JE, Fagents SA, Swanson DA (2012). Vesiculation of high fountaining Hawaiian eruptions: episodes 15 and 16 of 1959 Kilauea Iki. Bull. Volcanol..

[13] Mangan MT, Cashman KV (1996). The structure of basaltic scoria and reticulite and inferences for vesiculation, foam formation, and fragmentation in lava fountains. J. Volcanol. Geotherm. Res..

[14] Gonnermann HM (2015). Magma fragmentation. Annu. Rev. Earth Planet. Sci..

[15] Parcheta CE, Houghton BF, Swanson DA (2013). Contrasting patterns of vesiculation in low, intermediate, and high Hawaiian fountains: a case study of the 1969 Mauna Ulu eruption. J. Volcanol. Geotherm. Res..

[16] Namiki A, Patrick MR, Manga M, Houghton BF (2021). Brittle fragmentation by rapid gas separation in a Hawaiian fountain. Nat. Geosci..

[17] Stovall WK, Houghton BF, Gonnermann H, Fagents SA, Swanson DA (2011). Eruption dynamics of Hawaiian-style fountains: the case study of episode 1 of the Kilauea Iki 1959 eruption. Bull. Volcanol..

[18] Houghton BF, Gonnermann HM (2008). Basaltic explosive volcanism: constraints from deposits and models. Geochemistry.

[19] Cannata CB (2019). First 3D imaging characterization of Pele’s hair from Kilauea volcano (Hawaii). Sci. Rep..

[20] Jones TJ, Houghton BF, Llewellin EW, Parcheta CE, Höltgen L (2018). Spatter matters—distinguishing primary (eruptive) and secondary (non-eruptive) spatter deposits. Sci. Rep..

[21] Shimozuru D (1994). Physical parameters governing the formation of Pele’s hair and tears. Bull. Volcanol..

[22] Stoppa F (1996). The San Venanzo maar and tuff ring, Umbria, Italy: eruptive behaviour of a carbonatite-melilitite volcano. Bull. Volcanol..

[23] Junqueira-Brod TC, Brod JA, Thompson RN, Gibson SA (1999). Spinning droplets—a conspicuous lapilli-size structure in kamafugitic diatremes of Southern Goiás, Brazil. Braz. J. Geol..

[24] Lloyd FE, Stoppa F (2003). Pelletal lapilli in diatremes—some inspiration from the old masters. Geolines.

[25] Stoppa F, Woolley AR, Cundari A (2002). Extension of the melilite-carbonatite province in the Apennines of Italy: the kamafugite of Grotta del Cervo, Abruzzo. Mineral. Mag..

[26] Mitchell, R. H. *Kimberlites, Orangeites, and Related Rocks* 1–90 (Springer, 1995).

[27] Bosshard-Stadlin SA, Mattsson HB, Keller J (2014). Magma mixing and forced exsolution of CO2 during the explosive 2007–2008 eruption of Oldoinyo Lengai (Tanzania). J. Volcanol. Geotherm. Res..

[28] Gernon TM, Brown RJ, Tait MA, Hincks TK (2012). The origin of pelletal lapilli in explosive kimberlite eruptions. Nat. Commun..

[29] Wilson L, Head JW (2007). An integrated model of kimberlite ascent and eruption. Nature.

[30] Stoppa F, Lloyd FE, Tranquilli A, Schiazza M (2011). Comment on: Development of spheroid “composite” bombs by welding of juvenile spinning and isotropic droplets inside a mafic “eruption” column by Carracedo Sánchez et al.(2009). J. Volcanol. Geotherm. Res..

[31] Sánchez MC, Arostegui J, Sarrionandia F, Larrondo E, Ibarguchi JIG (2010). Cryptoachneliths: Hidden glassy ash in composite spheroidal lapilli. J. Volcanol. Geotherm. Res..

[32] Sánchez MC, Sarrionandia F, Arostegui J, Larrondo E, Ibarguchi JIG (2009). Development of spheroidal composite bombs by welding of juvenile spinning and isotropic droplets inside a mafic eruption column. J. Volcanol. Geotherm. Res..

[33] Di Piazza A (2017). Like a cannonball: origin of dense spherical basaltic ejecta. Bull. Volcanol..

[34] Brady LF, Webb RW (1943). Cored bombs from Arizona and California volcanic cones. J. Geol..

[35] Sottili G, Taddeucci J, Palladino DM (2010). Constraints on magma–wall rock thermal interaction during explosive eruptions from textural analysis of cored bombs. J. Volcanol. Geotherm. Res..

[36] Rosseel, J.-B., White, J. D. L. & Houghton, B. F. Complex bombs of phreatomagmatic eruptions: role of agglomeration and welding in vents of the 1886 Rotomahana eruption, Tarawera, New Zealand. *J. Geophys. Res. Solid Earth*10.1029/2005JB004073 (2006).

[37] Mueller SB, Houghton BF, Swanson DA, Poret M, Fagents SA (2019). Total grain size distribution of an intense Hawaiian fountaining event: case study of the 1959 Kilauea Iki eruption. Bull. Volcanol..

[38] Pioli L, Harris AJL (2019). Real-time geophysical monitoring of particle size distribution during volcanic explosions at stromboli Volcano (Italy). Front. Earth Sci..

[39] Brown RJ, Thordarson T, Self S, Blake S (2015). Disruption of tephra fall deposits caused by lava flows during basaltic eruptions. Bull. Volcanol..

[40] Taddeucci J (2021). Fracturing and healing of basaltic magmas during explosive volcanic eruptions. Nat. Geosci..

[41] Edwards MJ, Pioli L, Harris AJL, Gurioli L, Thivet S (2020). Magma fragmentation and particle size distributions in low intensity mafic explosions: the July/August 2015 Piton de la Fournaise eruption. Sci. Rep..

[42] Bombrun M, Harris A, Gurioli L, Battaglia J, Barra V (2015). Anatomy of a Strombolian eruption: inferences from particle data recorded with thermal video. J. Geophys. Res. Solid Earth.

[43] Melosh HJ, Vickery AM (1991). Melt droplet formation in energetic impact events. Nature.

[44] Martin PG (2020). Structural and compositional characteristics of Fukushima release particulate material from Units 1 and 3 elucidates release mechanisms, accident chronology and future decommissioning strategy. Sci. Rep..

[45] Dawson JB (1964). Carbonate tuff cones in northern Tanganyika. Geol. Mag..

[46] Haddock D (2020). Syn-eruptive agglutination of kimberlite volcanic ash. Volcanica.

[47] Shaikh AM, Tappe S, Bussweiler Y, Vollmer C, Brown RJ (2021). Origins of olivine in Earth’s youngest kimberlite: Igwisi Hills volcanoes, Tanzania craton. Contrib. Mineral. Petrol..

[48] Willcox A (2015). Petrology, geochemistry and low-temperature alteration of lavas and pyroclastic rocks of the kimberlitic Igwisi Hills volcanoes, Tanzania. Chem. Geol..

[49] Jones TJ, Russell JK, Porritt LA, Brown RJ (2014). Morphology and surface features of olivine in kimberlite: implications for ascent processes. Solid Earth.

[50] Brett RC, Russell JK, Andrews GDM, Jones TJ (2015). The ascent of kimberlite: insights from olivine. Earth Planet. Sci. Lett..

[51] Liu EJ, Cashman KV, Rust AC (2015). Optimising shape analysis to quantify volcanic ash morphology. GeoResJ.

[52] Arndt NT (2010). Olivine, and the origin of kimberlite. J. Petrol..

[53] Jones TJ, Russell JK (2018). Attrition in the kimberlite system. Mineral. Petrol..

[54] Jones TJ, Russell JK, Sasse D (2019). Modification of mantle cargo by turbulent ascent of kimberlite. Front. Earth Sci..

[55] Sasse D, Jones TJ, Russell JK (2020). Transport, survival and modification of xenoliths and xenocrysts from source to surface. Earth Planet. Sci. Lett..

[56] Bindeman IN (2005). Fragmentation phenomena in populations of magmatic crystals. Am. Mineral..

[57] Villermaux E (2012). The formation of filamentary structures from molten silicates: Pele’s hair, angel hair, and blown clinker. CR Mécanique.

[58] Namiki A, Manga M (2008). Transition between fragmentation and permeable outgassing of low viscosity magmas. J. Volcanol. Geotherm. Res..

[59] Eggers J, Villermaux E (2008). Physics of liquid jets. Rep. Prog. Phys..

[60] Villermaux E (2007). Fragmentation. Annu. Rev. Fluid Mech..

[61] Marmottant P, Villermaux E (2004). On spray formation. J. Fluid Mech..

[62] Walker D, Mullins O (1981). Surface tension of natural silicate melts from 1200–1500 C and implications for melt structure. Contrib. Mineral. Petrol..

[63] Khitarov NI (1979). Effects of temperature, pressure, and volatiles on the surface tension of molten basalt. Geochem. Int..

[64] La Spina G (2021). Explosivity of basaltic lava fountains is controlled by magma rheology, ascent rate and outgassing. Earth Planet. Sci. Lett..

[65] Lasheras JC, Hopfinger EJ (2000). Liquid jet instability and atomization in a coaxial gas stream. Annu. Rev. Fluid Mech..

[66] Tadmor R (2017). Solid–liquid work of adhesion. Langmuir.

[67] Porritt LA, Russell JK (2012). Kimberlite ash: Fact or fiction. Phys. Chem. Earth Parts A/B/C..

[68] Panao MRO, Moreira ALN (2004). Experimental study of the flow regimes resulting from the impact of an intermittent gasoline spray. Exp. Fluids.

[69] Heine M (2013). Modeling of the spray zone for particle wetting in a fluidized bed. Chem. Ing. Tech..

[70] Bai C, Gosman AD (1995). Development of methodology for spray impingement simulation. SAE Trans..

[71] Bai, C. X., Rusche, H. & Gosman, A. D. Modeling of gasoline spray impingement. *At. Sprays* **12**, 1–28 (2002).

[72] Wang, A.-B., Chen, C.-C. & Hwang, W.-C. *Drop-Surface Interactions* 303–306 (Springer, 2002).

[73] Jones TJ, Russell JK (2017). Ash production by attrition in volcanic conduits and plumes. Sci. Rep..

[74] Mueller SB, Lane SJ, Kueppers U (2015). Lab-scale ash production by abrasion and collision experiments of porous volcanic samples. J. Volcanol. Geotherm. Res..

[75] Jones TJ (2016). Primary and secondary fragmentation of crystal-bearing intermediate magma. J. Volcanol. Geotherm. Res..

[76] Meyer JD (1971). Glass crust on intratelluric phenocrysts in volcanic ash as a measure of eruptive violence. Bull. Volcanol..

[77] Carracedo-Sánchez M, Sarrionandia F, Arostegui J, Errandonea-Martin J, Gil-Ibarguchi JI (2016). Petrography and geochemistry of achnelithic tephra from Las Herrerias Volcano (Calatrava volcanic field, Spain): formation of nephelinitic achneliths and post-depositional glass alteration. J. Volcanol. Geotherm. Res..

[78] Heiken GH, McKay DS, Brown RW (1974). Lunar deposits of possible pyroclastic origin. Geochim. Cosmochim. Acta.

[79] Martin PG (2019). Provenance of uranium particulate contained within Fukushima Daiichi Nuclear Power Plant Unit 1 ejecta material. Nat. Commun..

[80] Adams CE, Farlow NH, Schell WR (1960). The compositions, structures and origins of radioactive fall-out particles. Geochim. Cosmochim. Acta.

[81] Russell JK, Porritt LA, Lavallée Y, Dingwell DB (2012). Kimberlite ascent by assimilation-fuelled buoyancy. Nature.

[82] Chepurov AA, Pokhilenko NP (2015). Experimental estimation of the Kimberlite melt viscosity. Dokl. Earth Sci..

[83] Sparks RSJ (2006). Dynamical constraints on kimberlite volcanism. J. Volcanol. Geotherm. Res..

[84] Mader HM, Llewellin EW, Mueller SP (2013). The rheology of two-phase magmas: a review and analysis. J. Volcanol. Geotherm. Res..

[85] Smith, B. H. S. et al. Kimberlite terminology and classification. In *Proceedings of 10th International Kimberlite Conference* 1–17 (Springer, 2013).

