# Supplementary material for: Melt stripping and agglutination of pyroclasts during the explosive eruption of low viscosity magmas
Source: Nat Commun. 2022 Feb 22;13:992. doi: 10.1038/s41467-022-28633-w (PMC8863896; doi:10.1038/s41467-022-28633-w)
Supplement: Supplementary file 2 — Description of Additional Supplementary Files [file 41467_2022_28633_MOESM2_ESM.pdf]

Description of Additional Supplementary Files

File Name: Supplementary Data 1
Description: An excel file containing all the raw image analysis data plotted in Figure 3. The file has four tabs, corresponding to the data plotted in the four figure panels a through d.
