# Supplementary material for: Melt stripping and agglutination of pyroclasts during the explosive eruption of low viscosity magmas
Source: Nat Commun. 2022 Feb 22;13:992. doi: 10.1038/s41467-022-28633-w (PMC8863896; doi:10.1038/s41467-022-28633-w)
Supplement: Supplementary file 1 — Supplementary Information [file 41467_2022_28633_MOESM1_ESM.pdf]

Supplementary Material to Accompany:

Melt stripping and agglutination of pyroclasts during the explosive eruption of low viscosity magmas

Thomas J. Jones, James K. Russell, Richard J. Brown, Lea Hollendonner

Included in this pdf file:

Figure S1
Description: Merged SEM images and accompanying image traces for all 30 pyroclasts analysed in this study. All scale bars measure 500 µm.

Figure S2
Description: Energy-dispersive X-ray spectroscopy (EDS) analysis from a line scan. The line position is marked on the SEM image and transects the olivine crystal, inner homogenous melt, and outer complex melt. These transitions from olivine to homogeneous melt and from homogeneous melt to complex melt are marked by black vertical dashed lines on the spectra. For all spectra, no chemical differences are observed between the homogeneous and complex melts (now glasses).

Available as a separate download:

File name: Supplementary Data 1.xlsx
Description: An excel file containing all the raw image analysis data plotted in Figure 3. The file has four tabs, corresponding to the data plotted in the four figure panels a through d.

Fig. S1. Merged SEM images and accompanying image traces for all 30 pyroclasts analyzed in this study. All scale bars measure 500 μm.

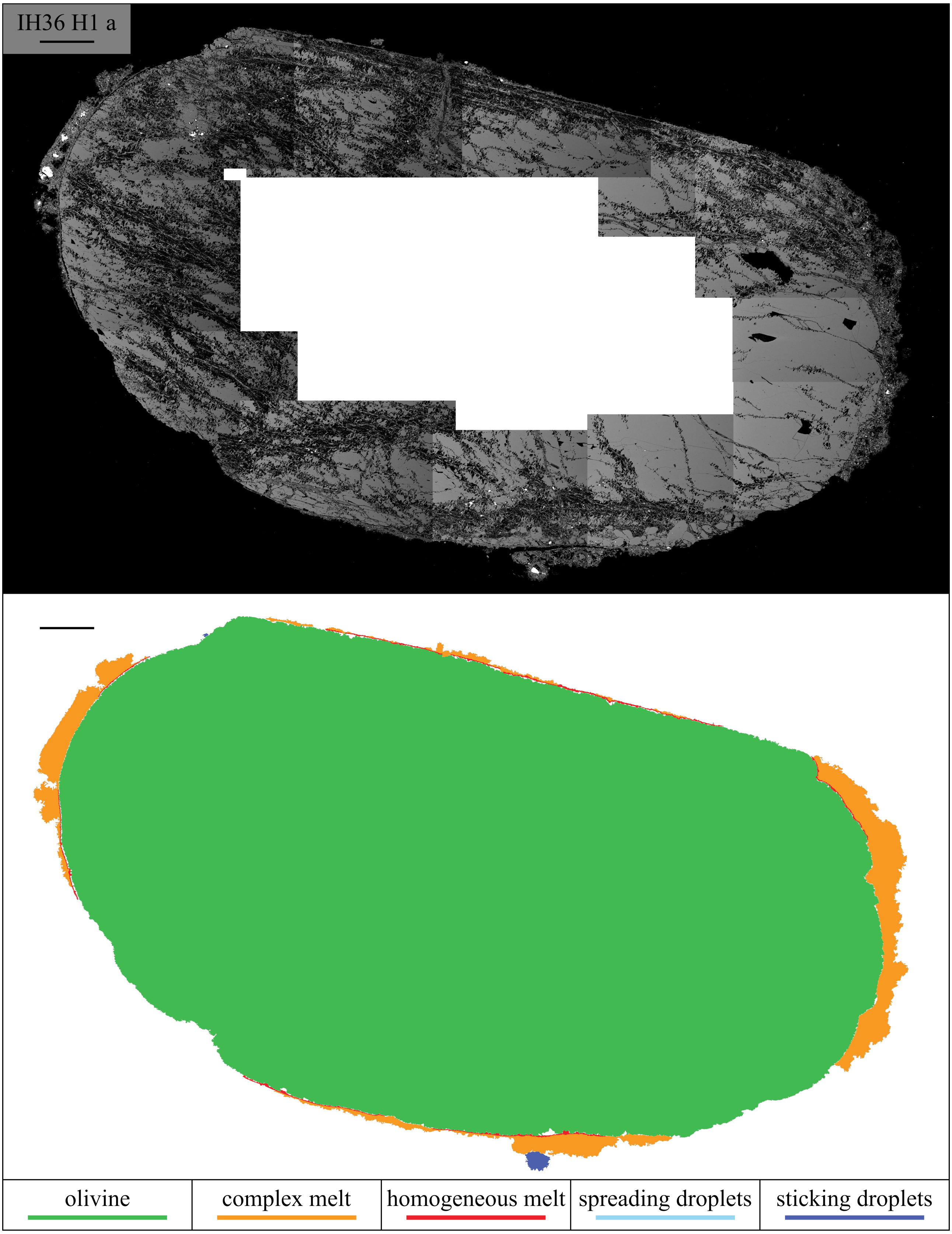

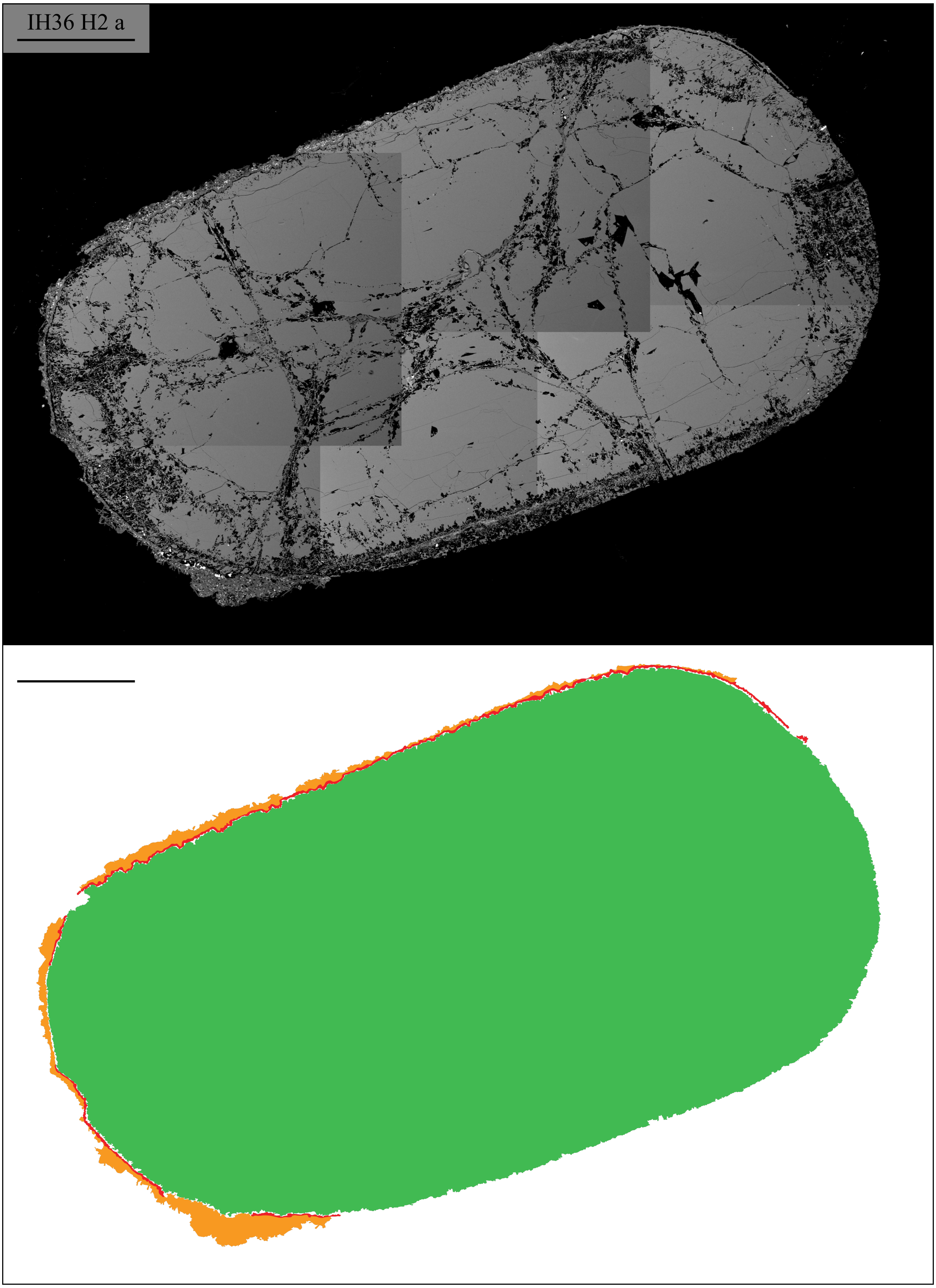
IH36 H2 a

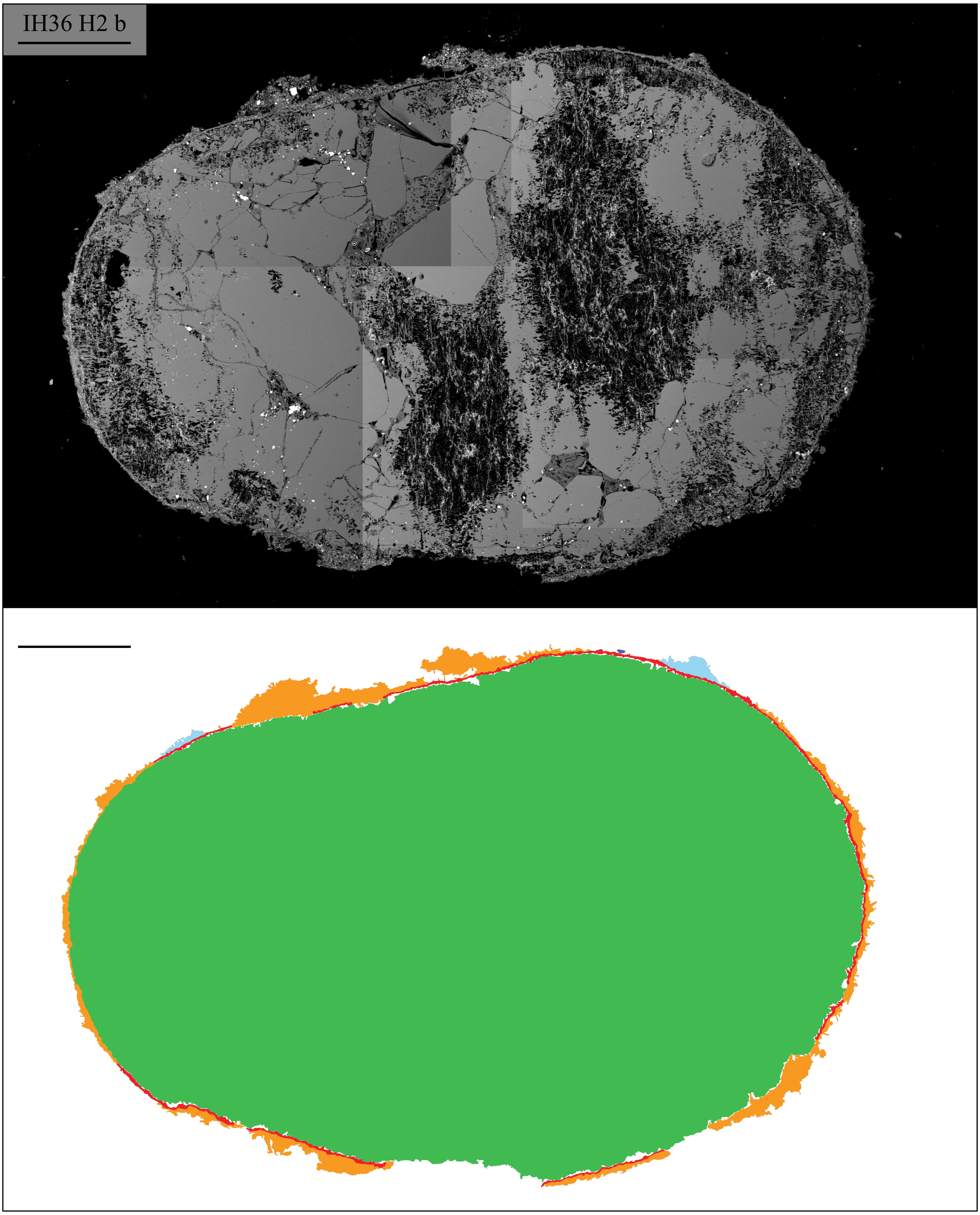
IH36 H2 b

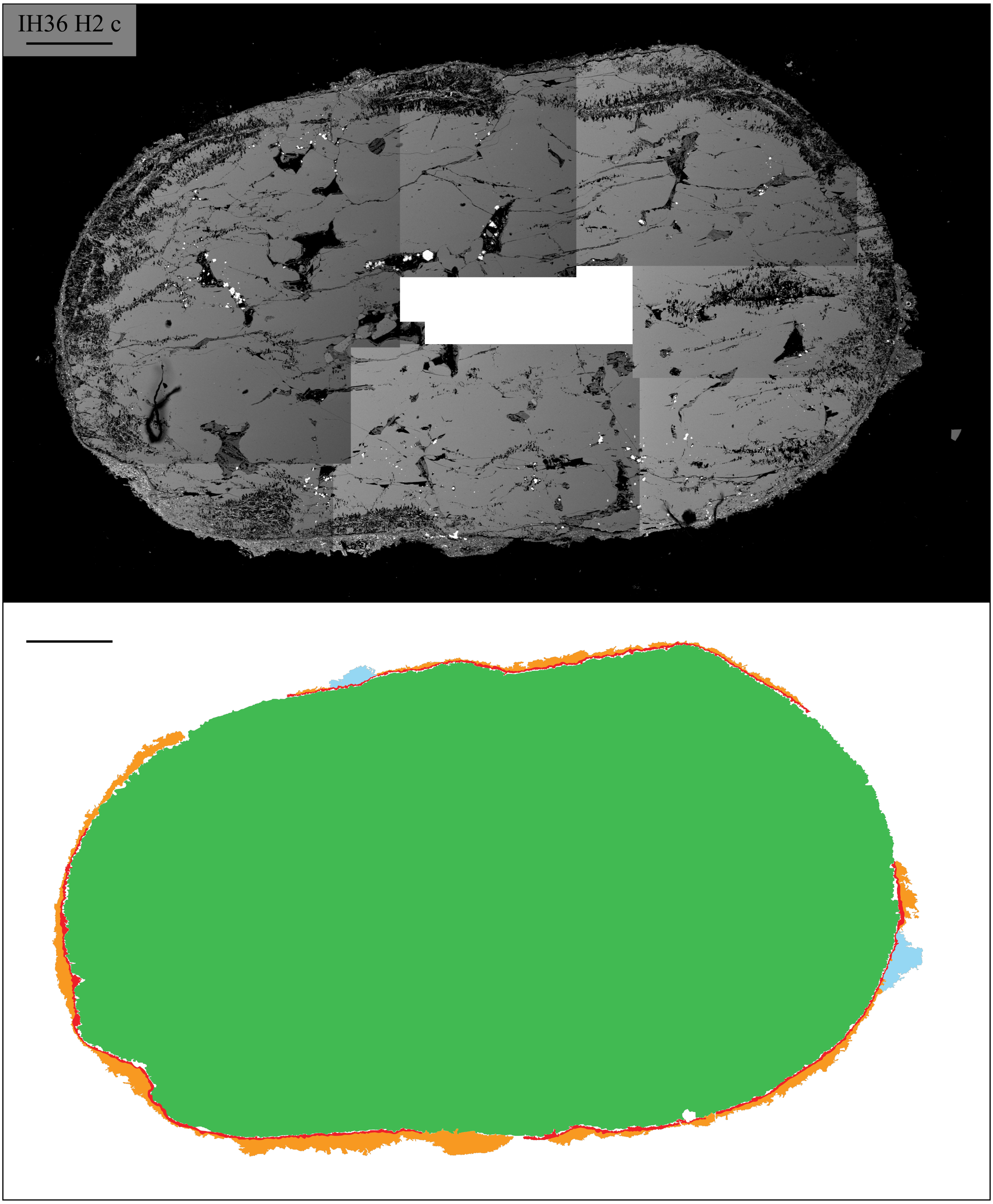
IH36 H2 c

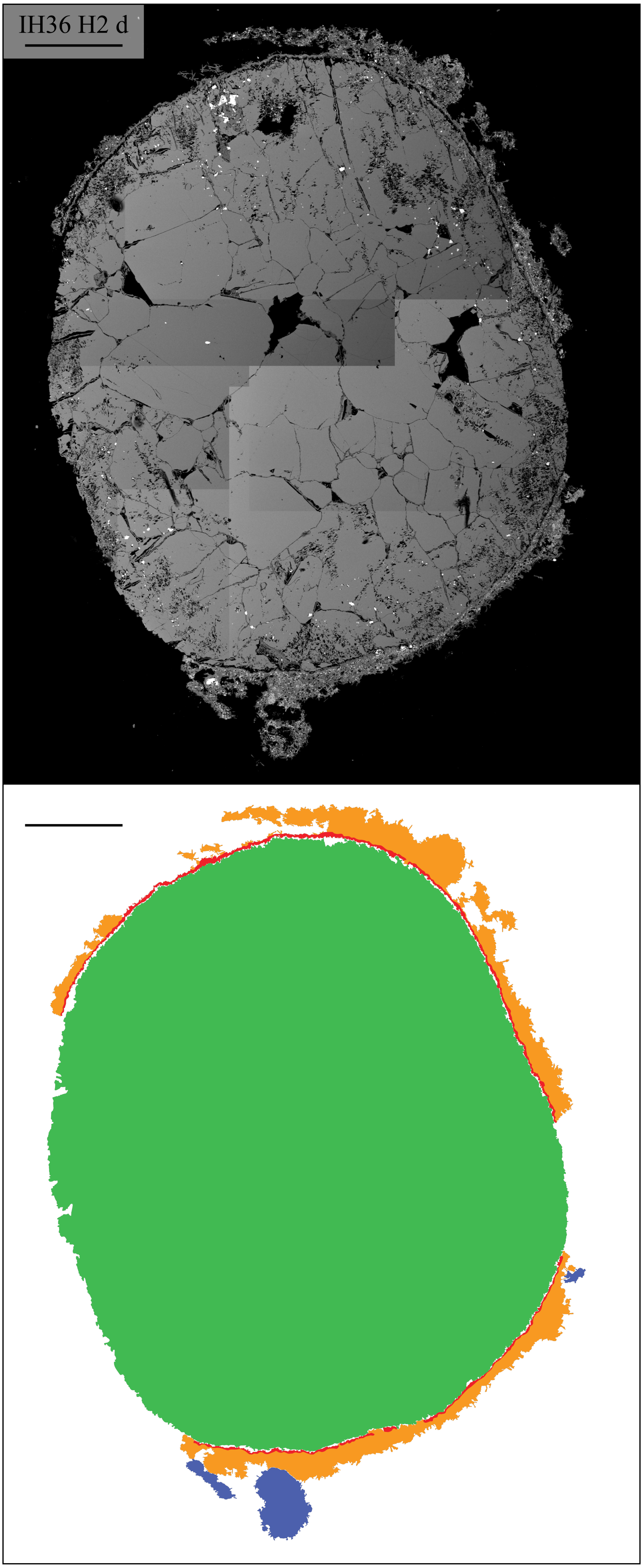
IH36 H2 d

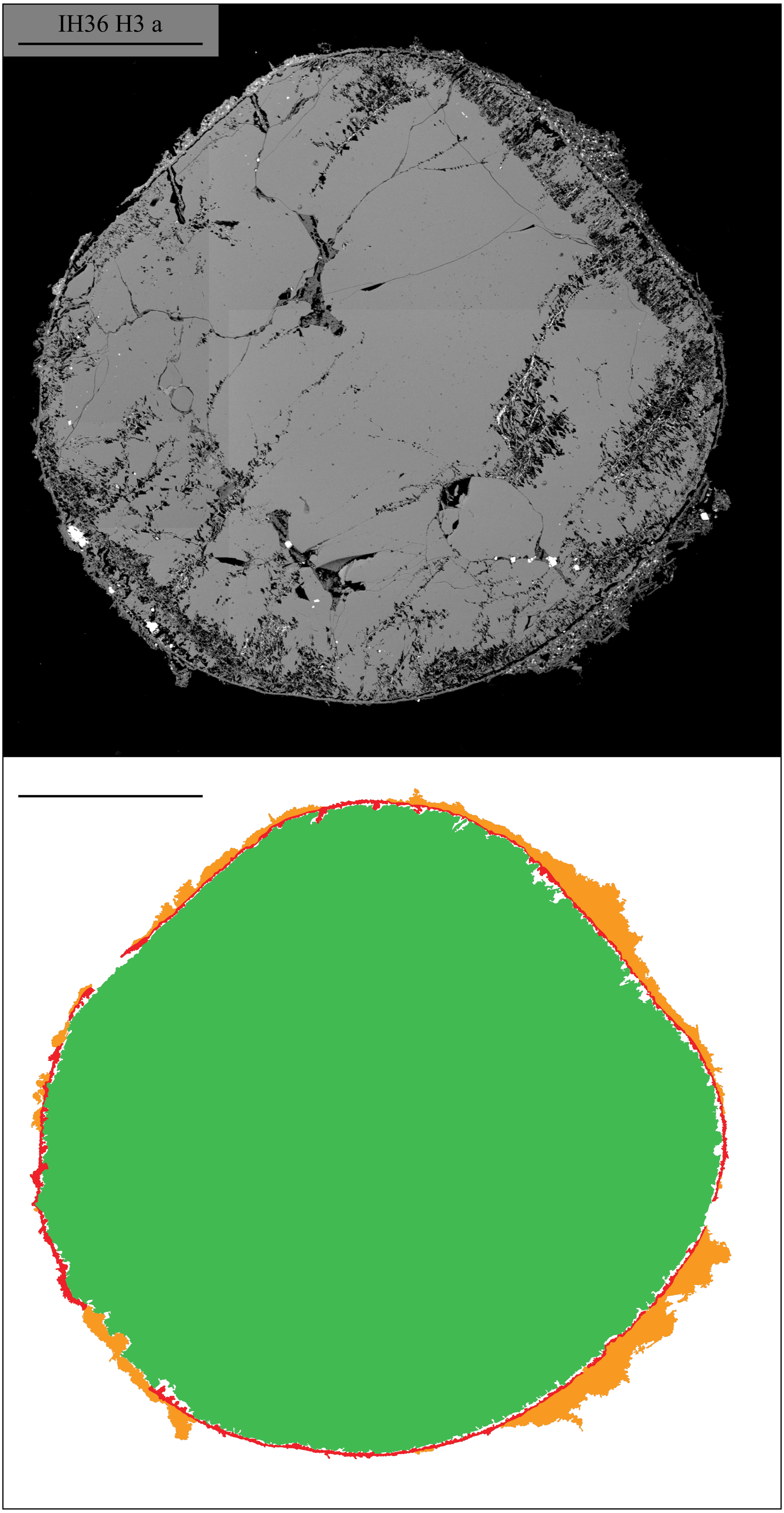
IH36 H3 a

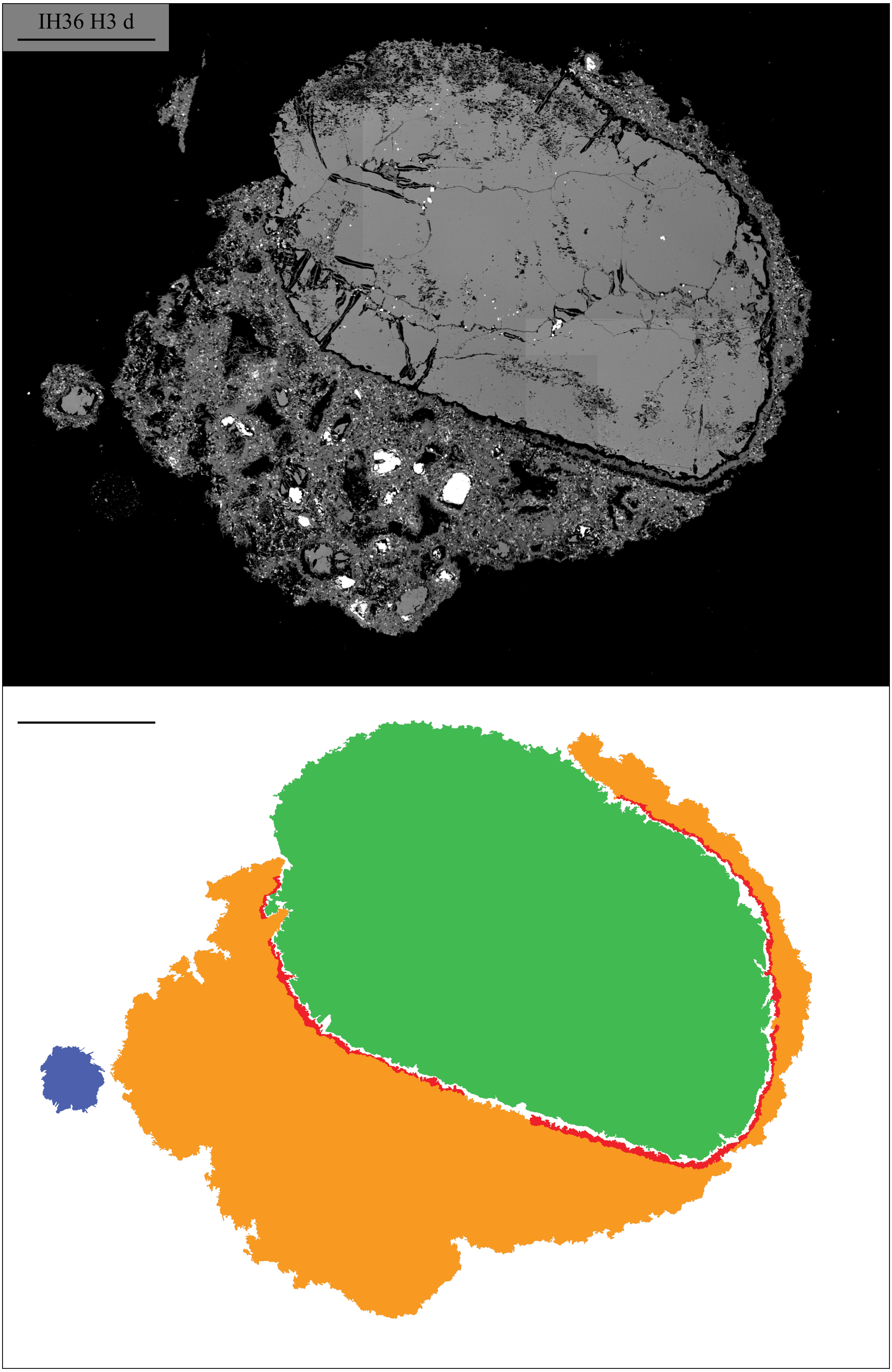
IH36 H3 d

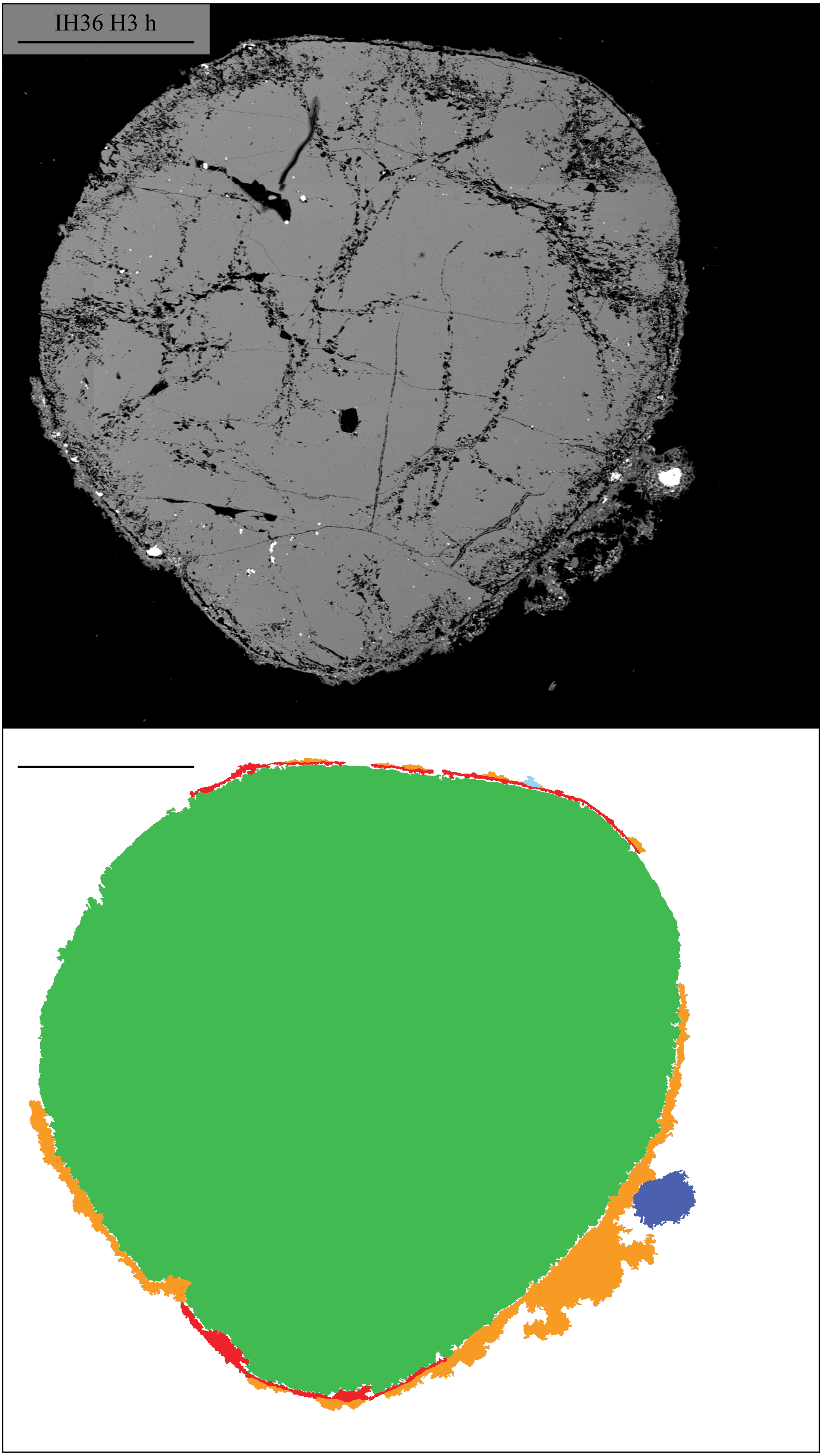
IH36 H3 h

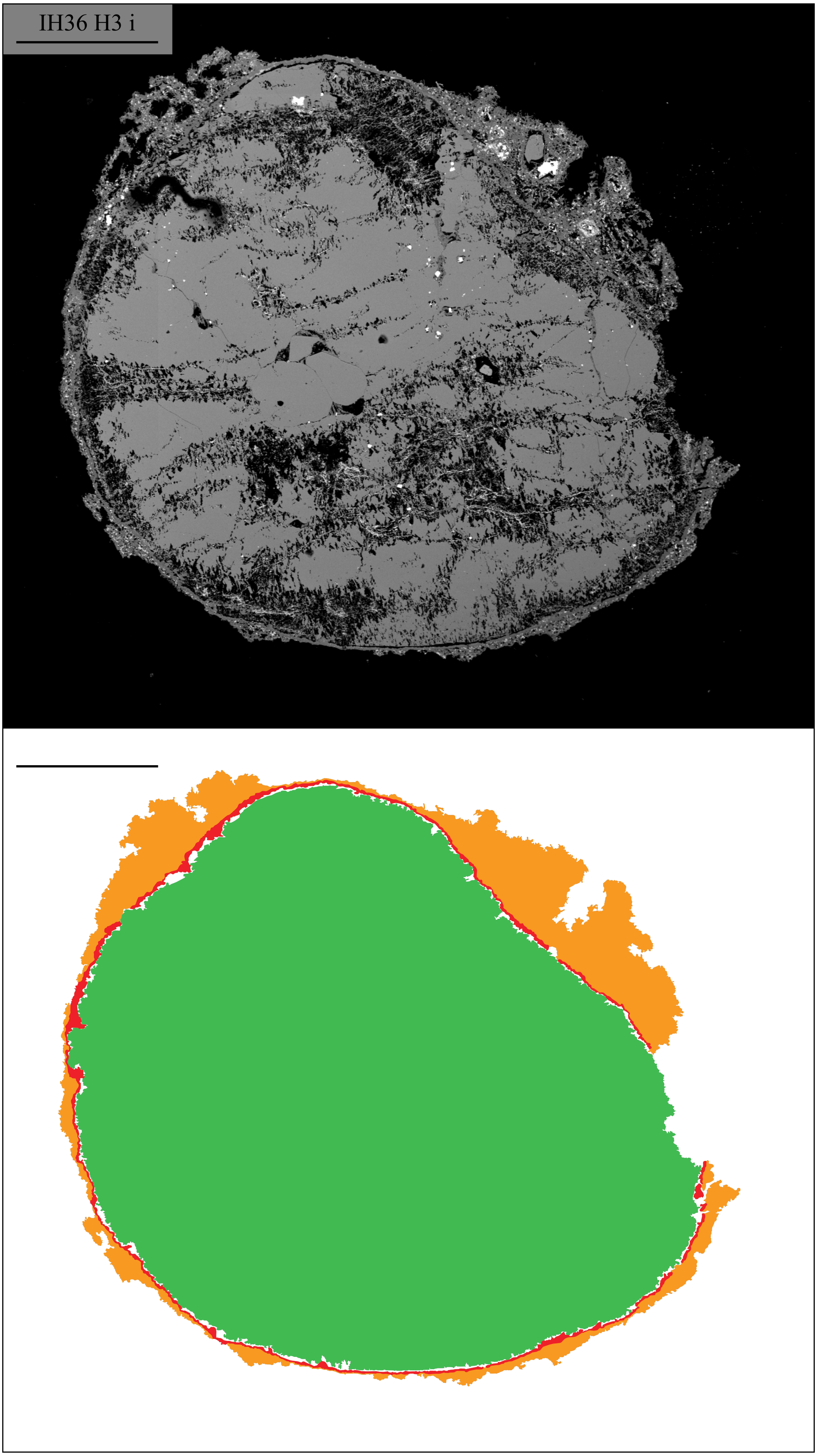
IH36 H3 i

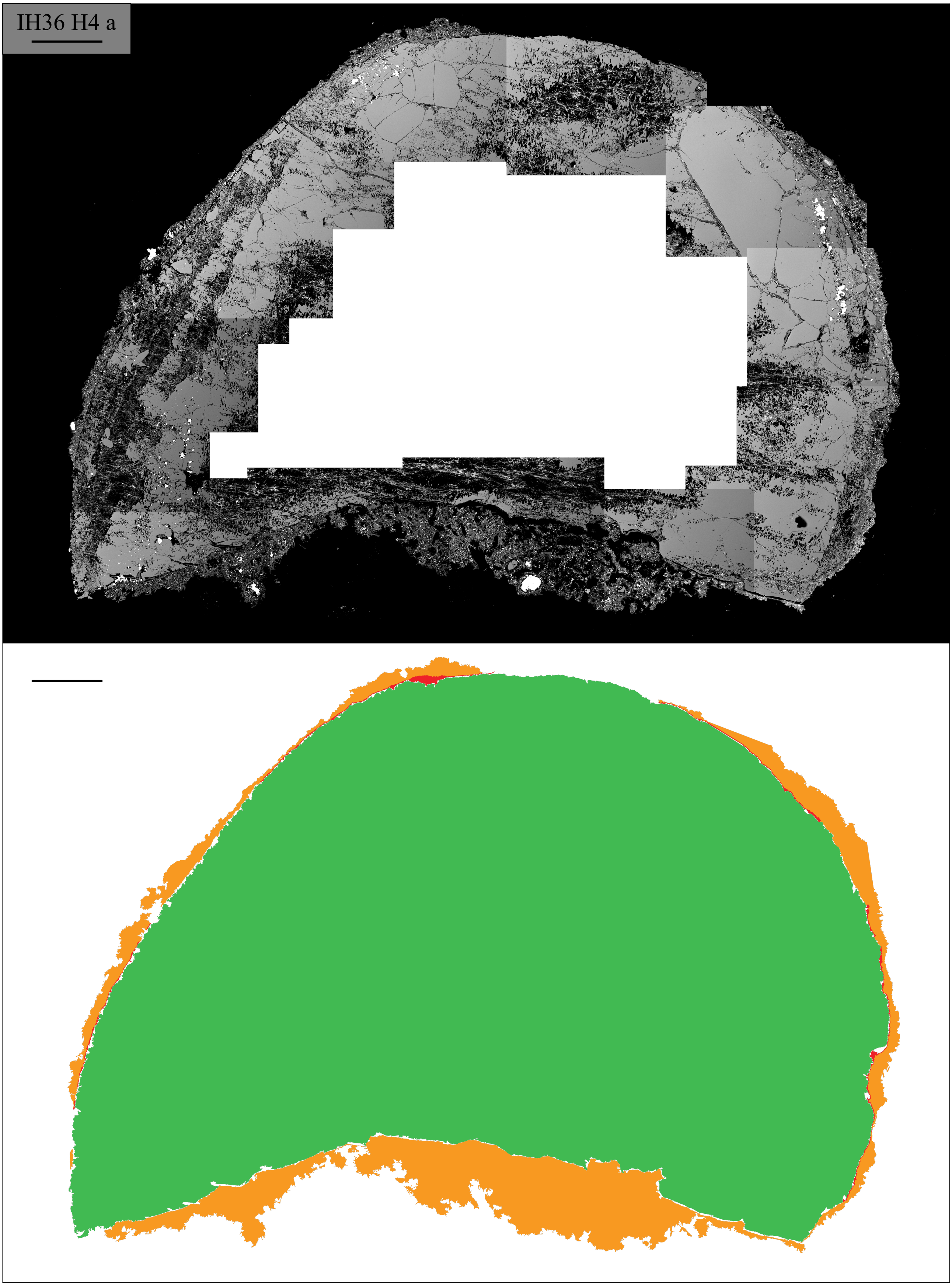
IH36 H4 a

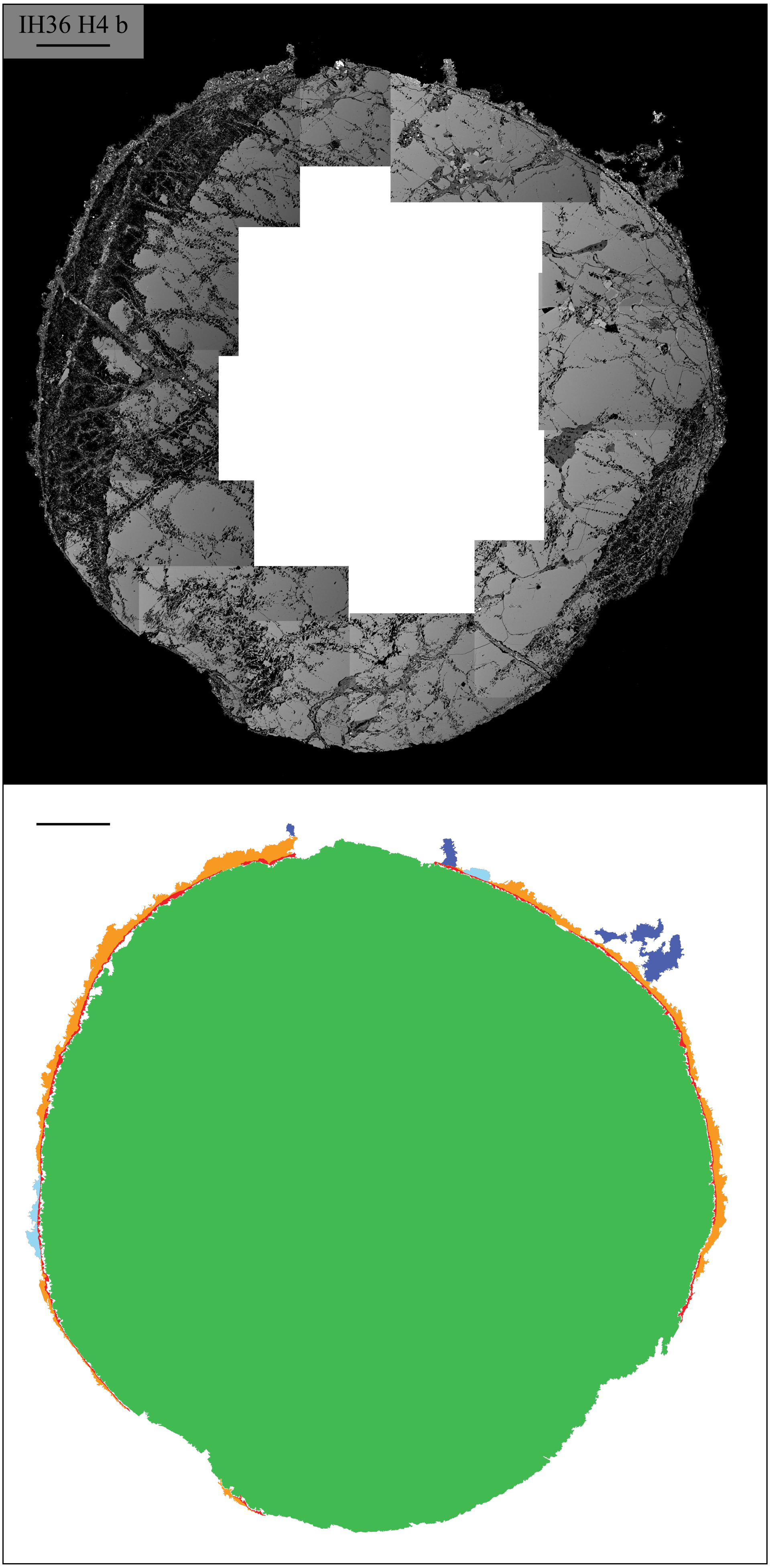
IH36 H4 b

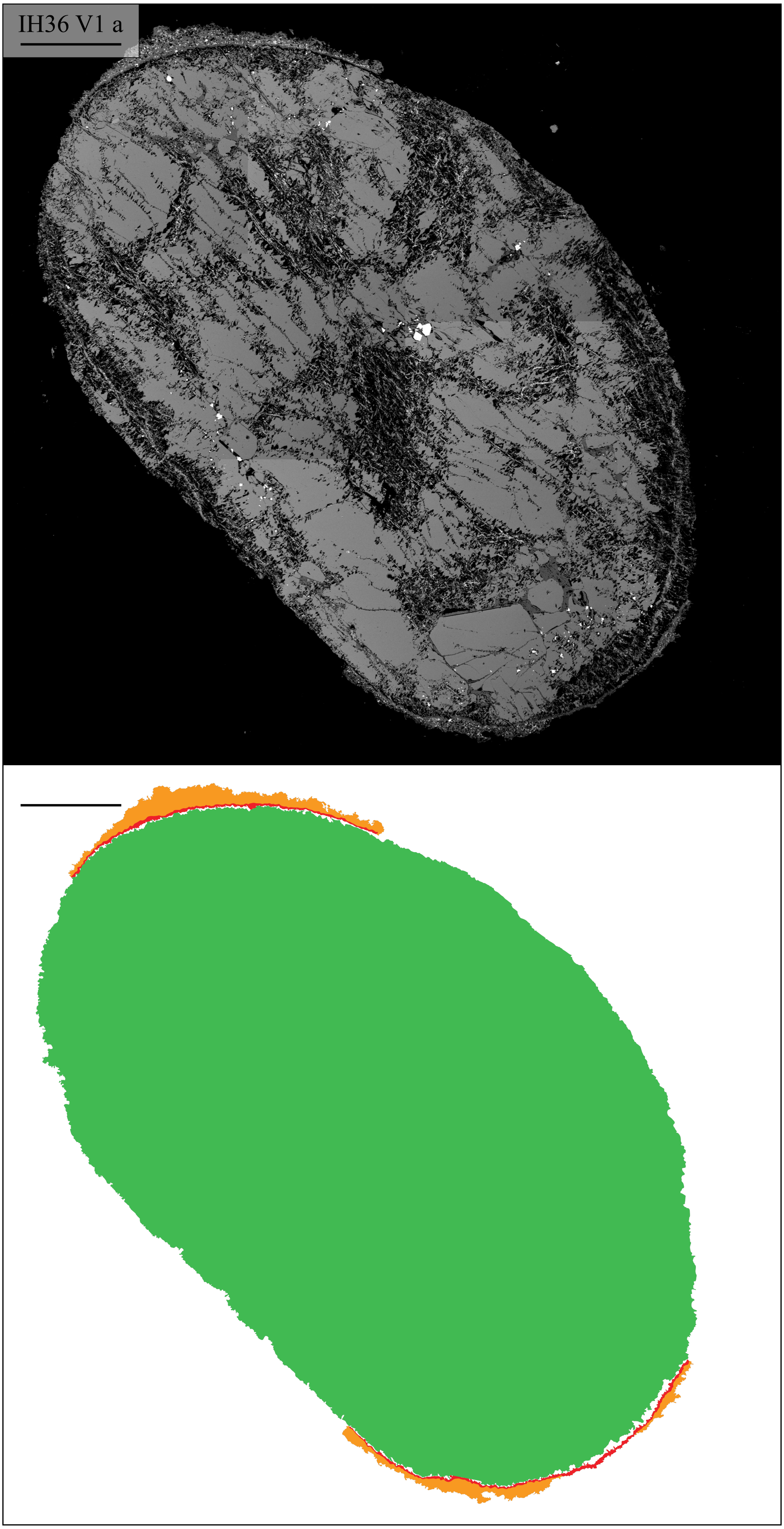
IH36 V1 a

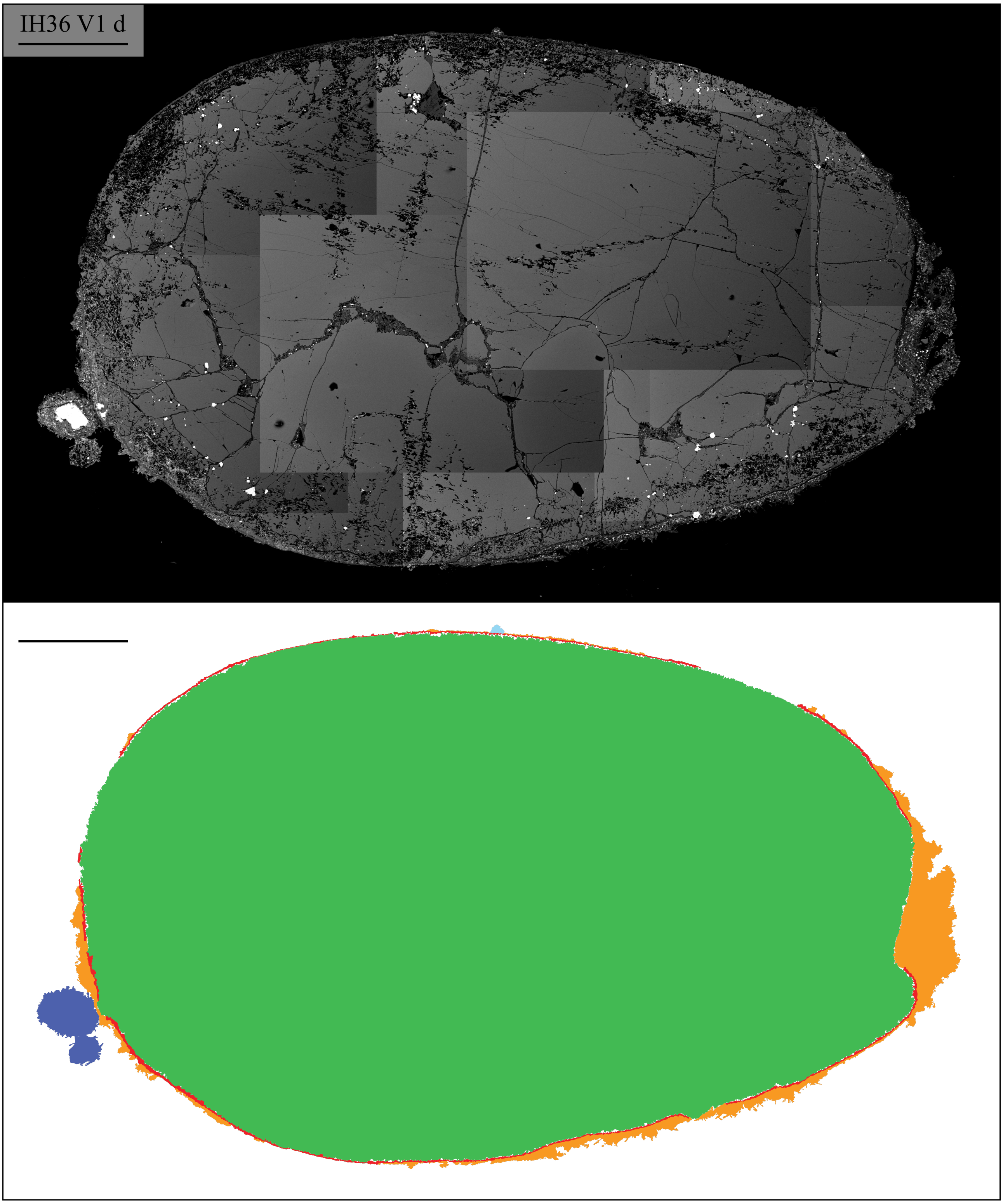
IH36 V1 d

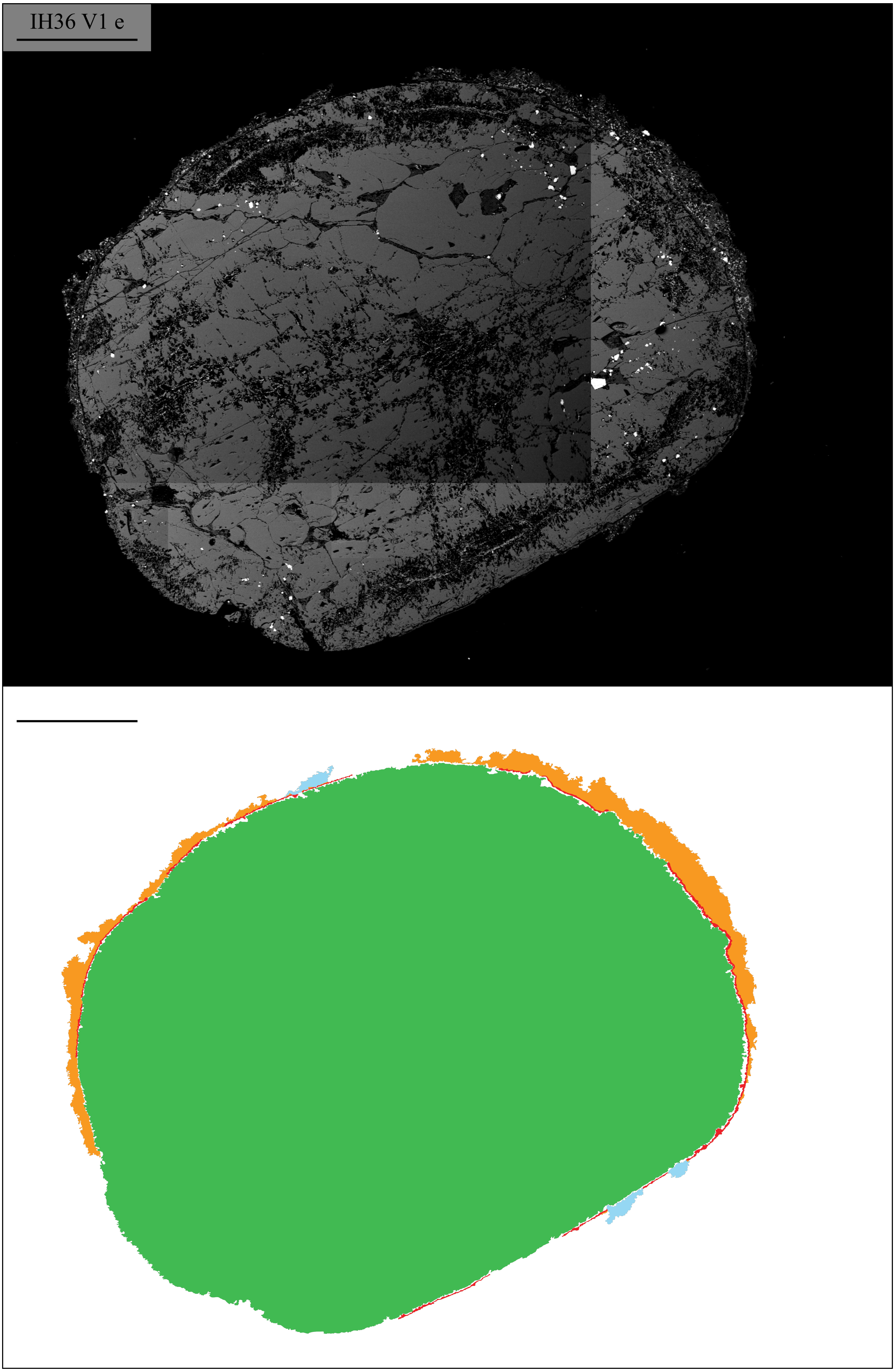
IH36 V1 e

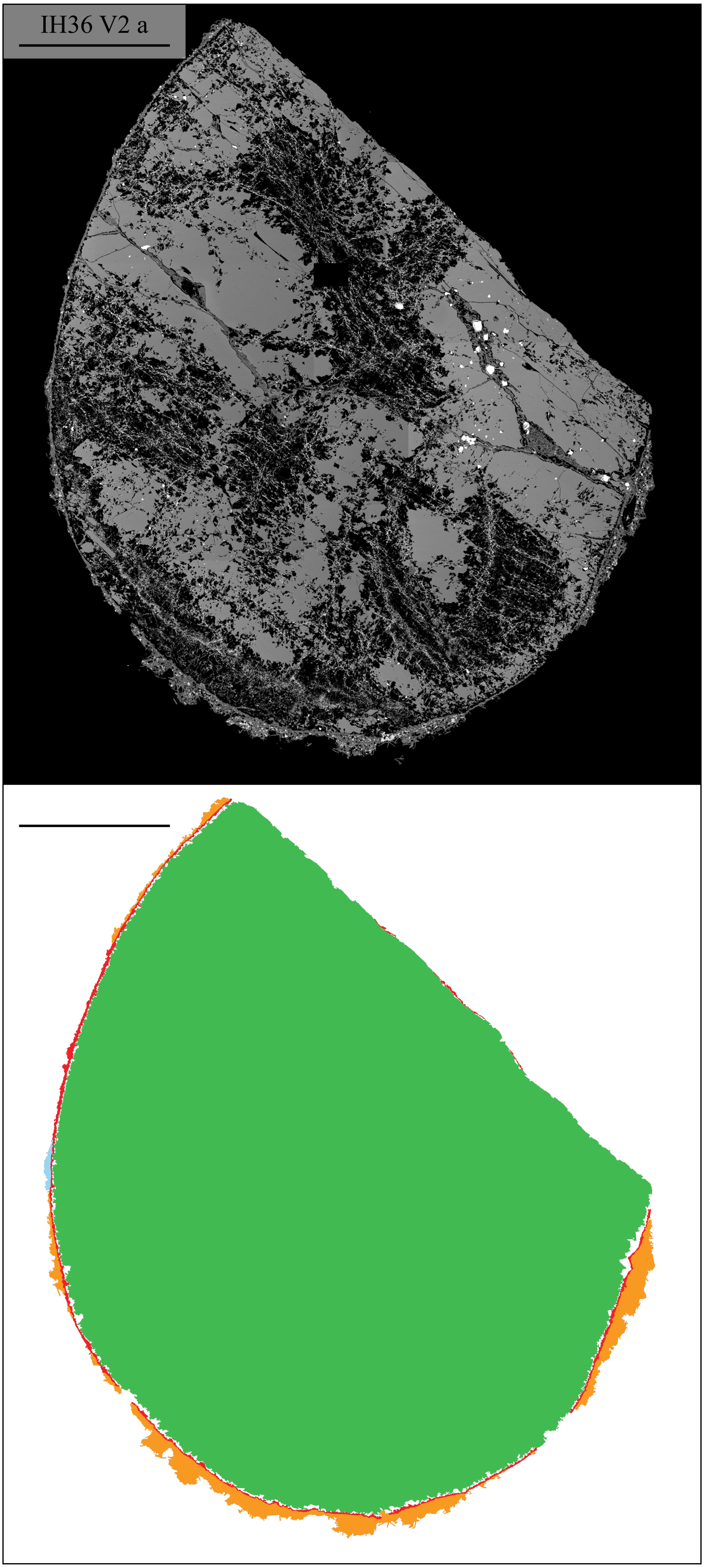
IH36 V2 a

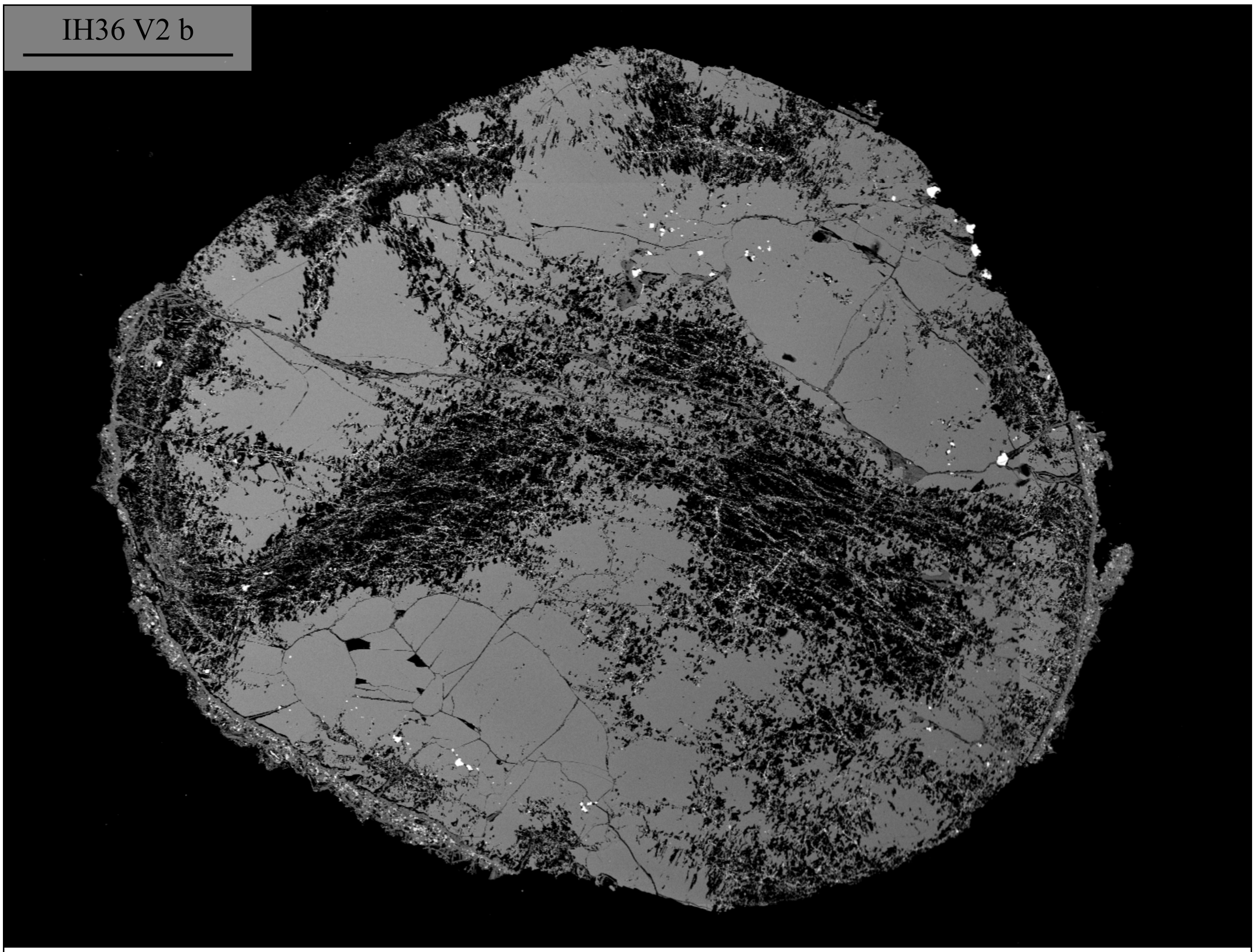
IH36 V2 b

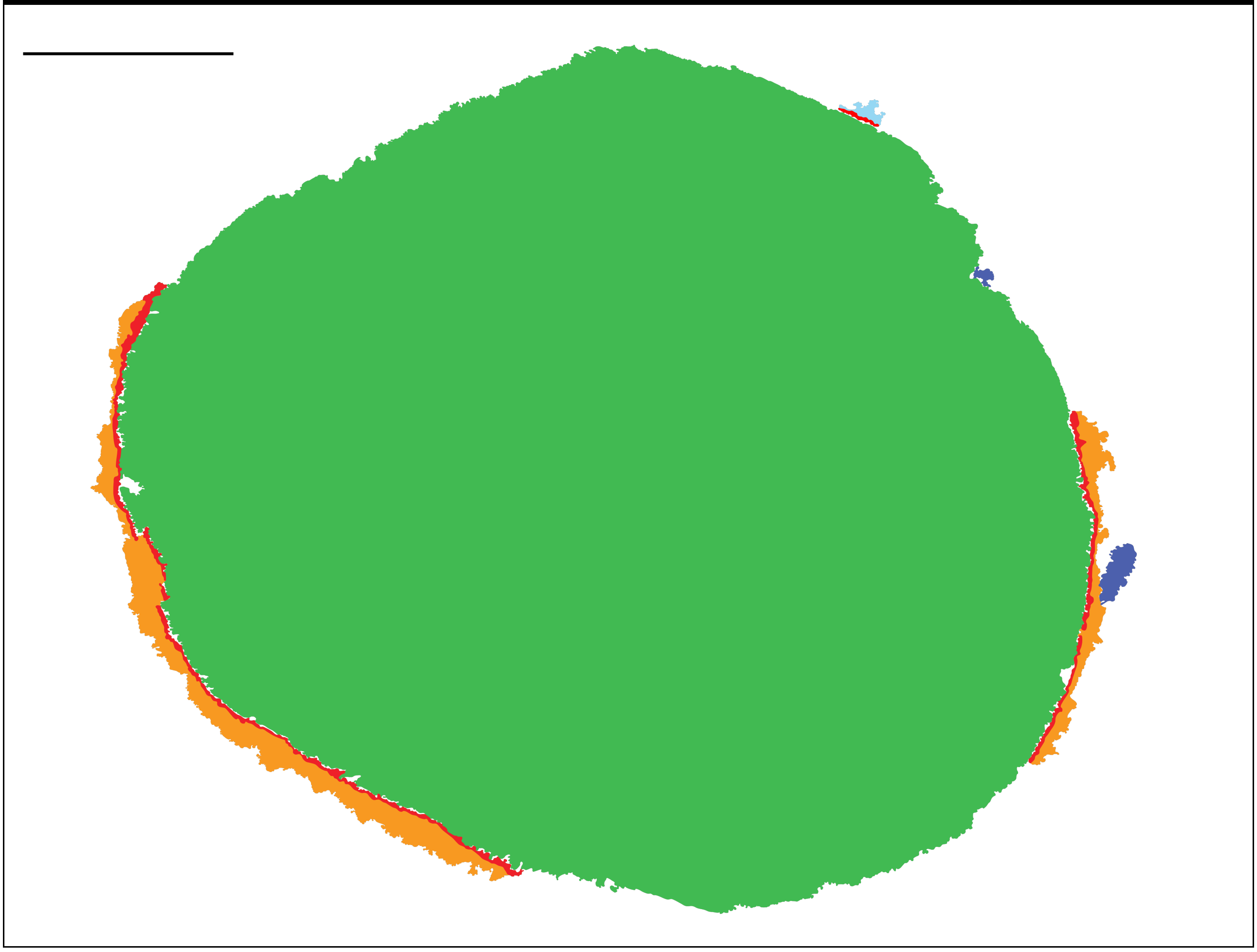

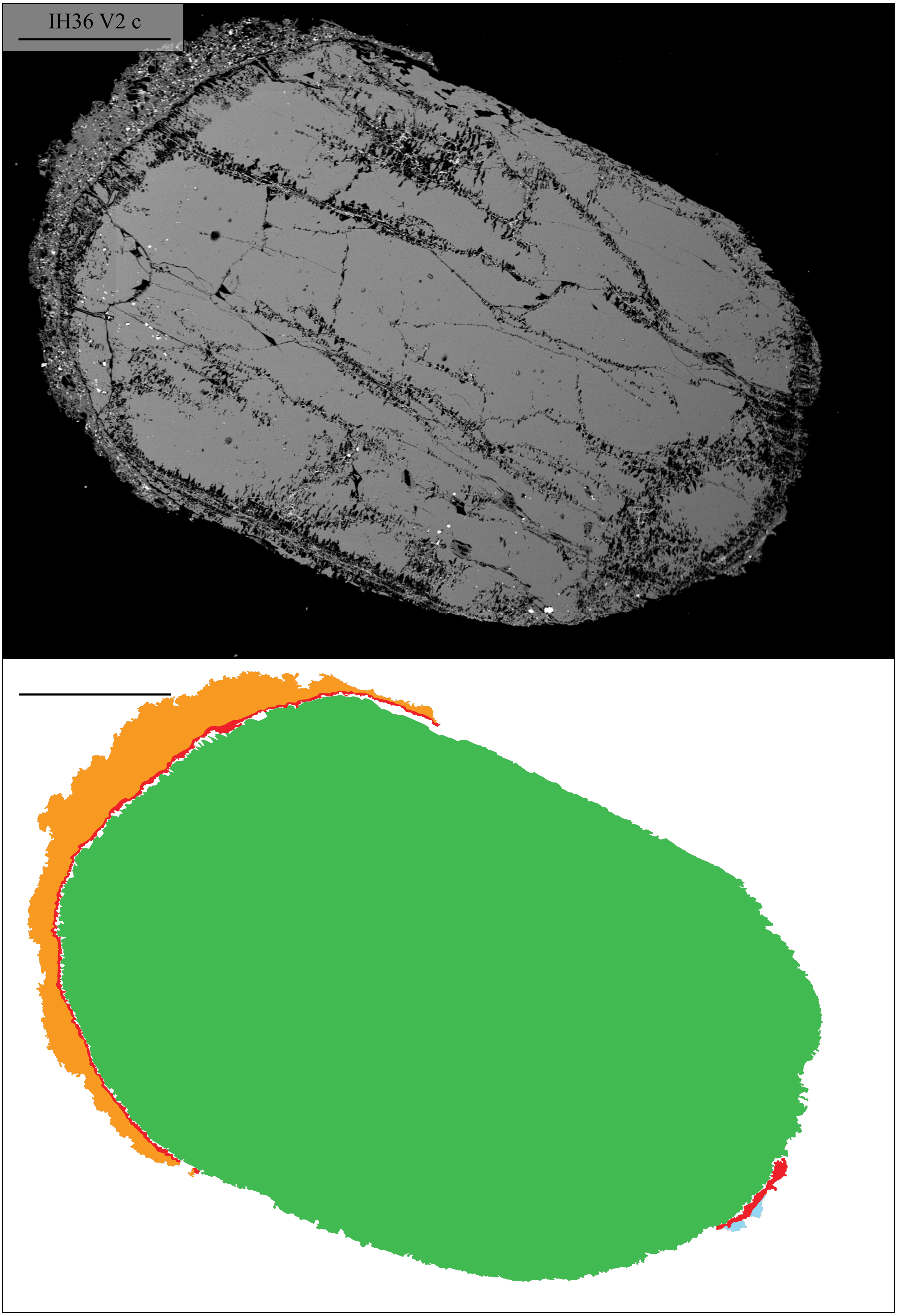
IH36 V2 c

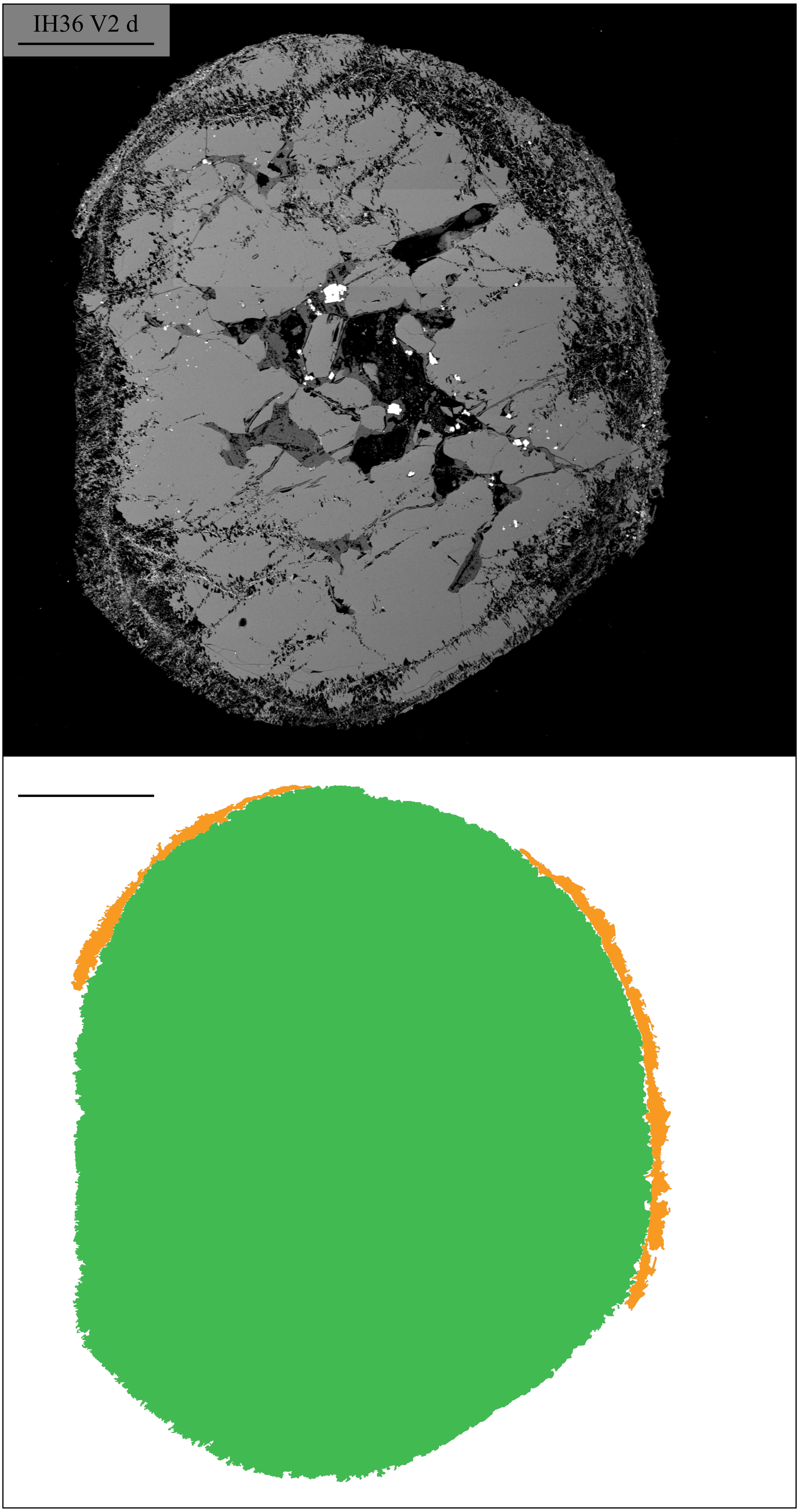
IH36 V2 d

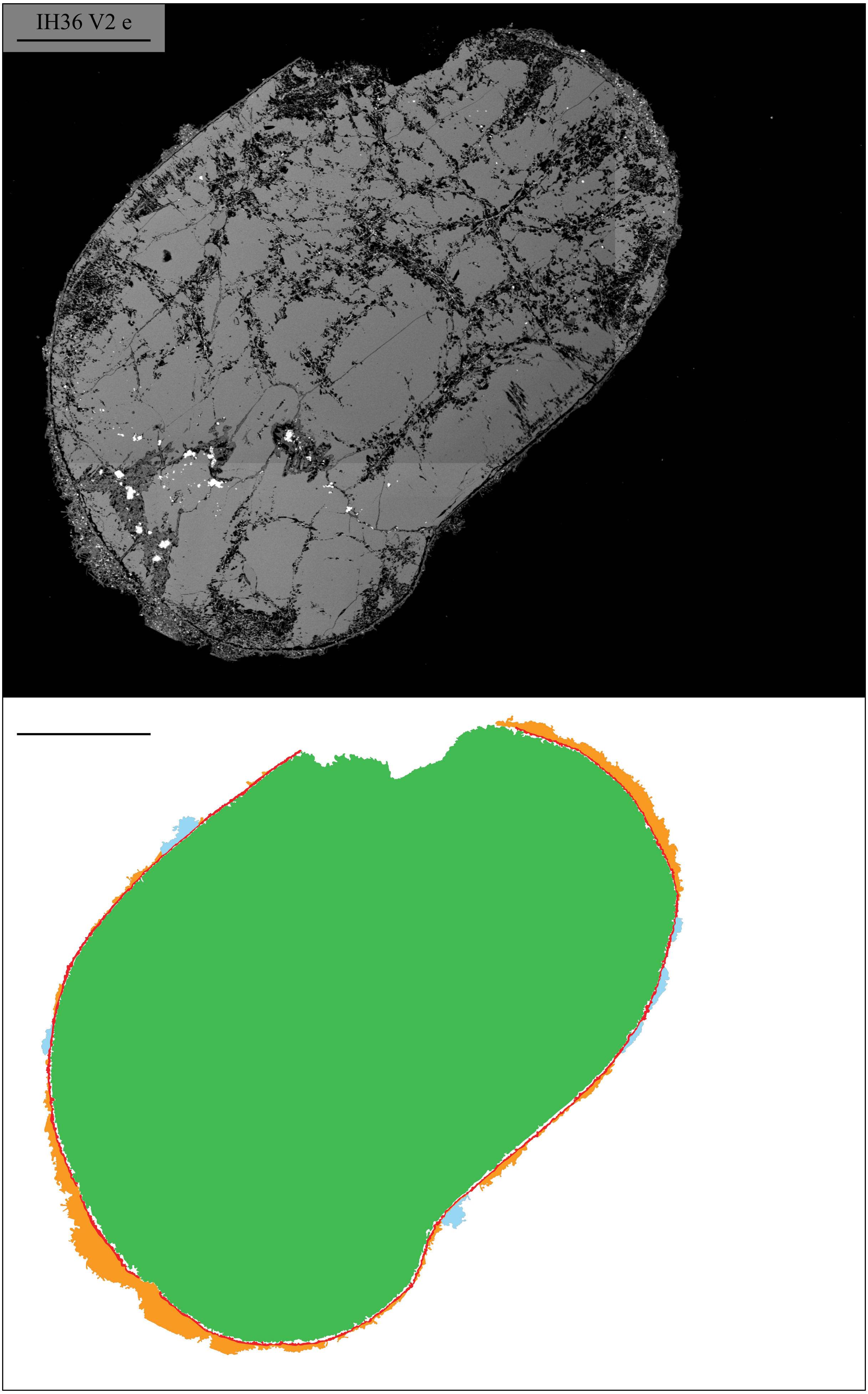
IH36 V2 e

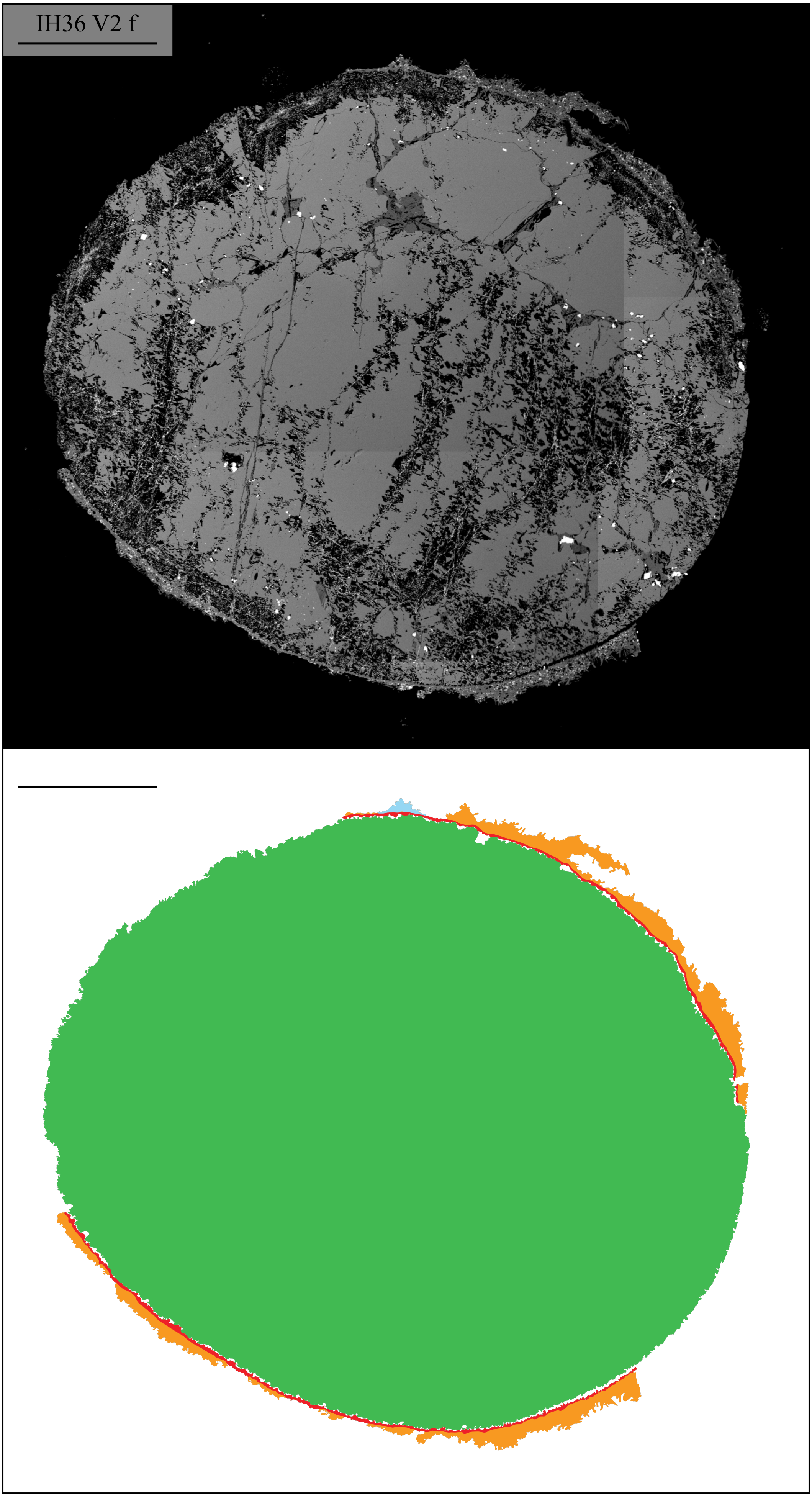
IH36 V2 f

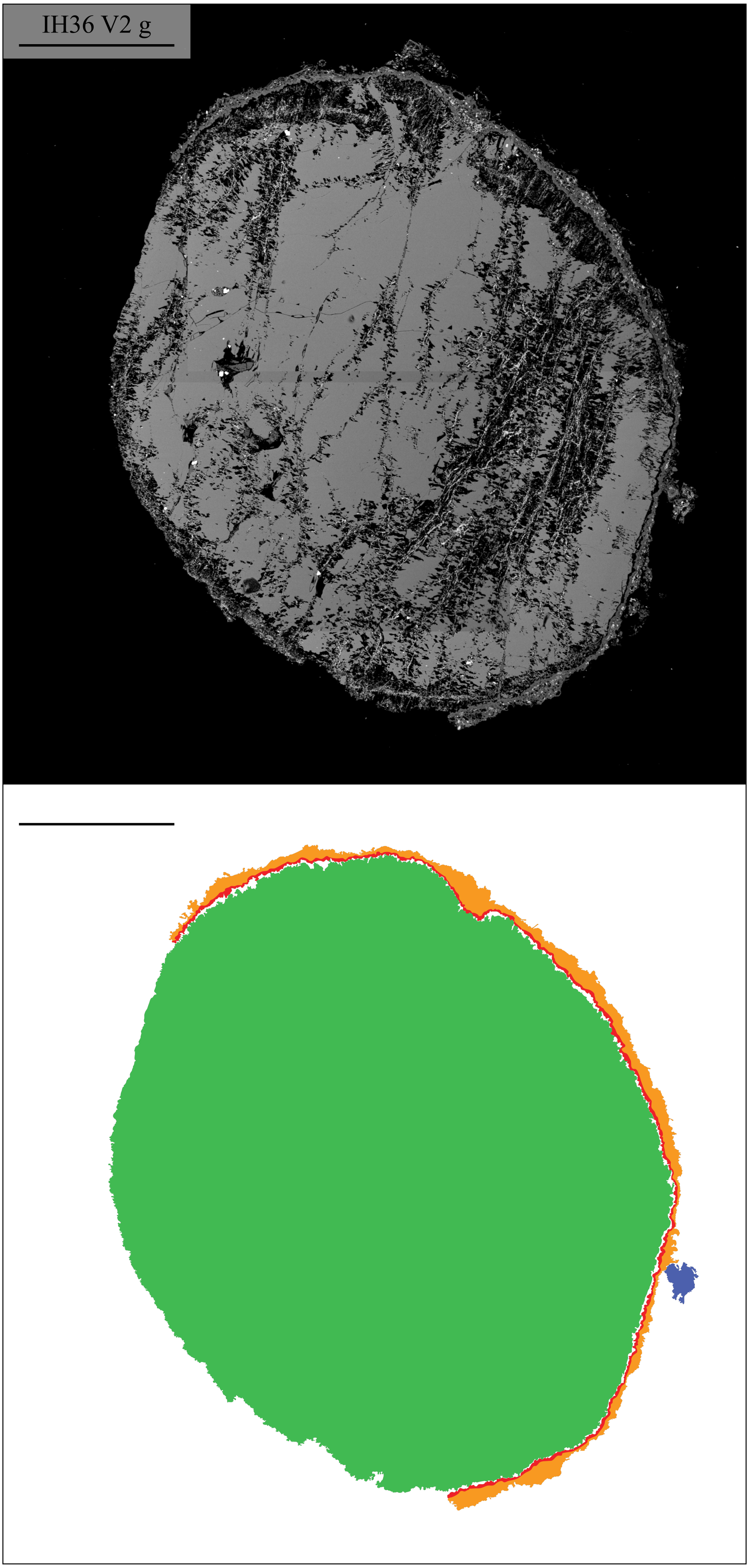
IH36 V2 g

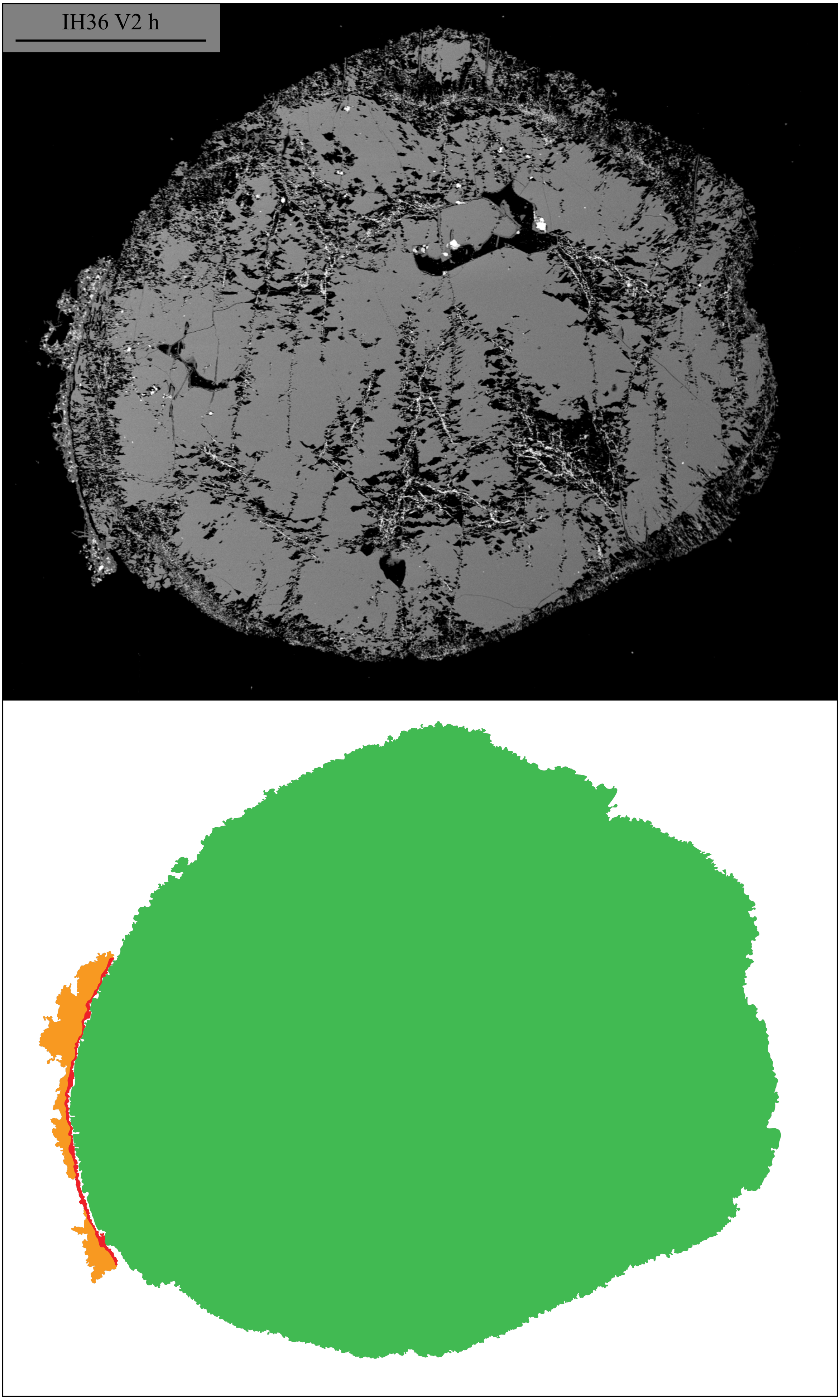
IH36 V2 h

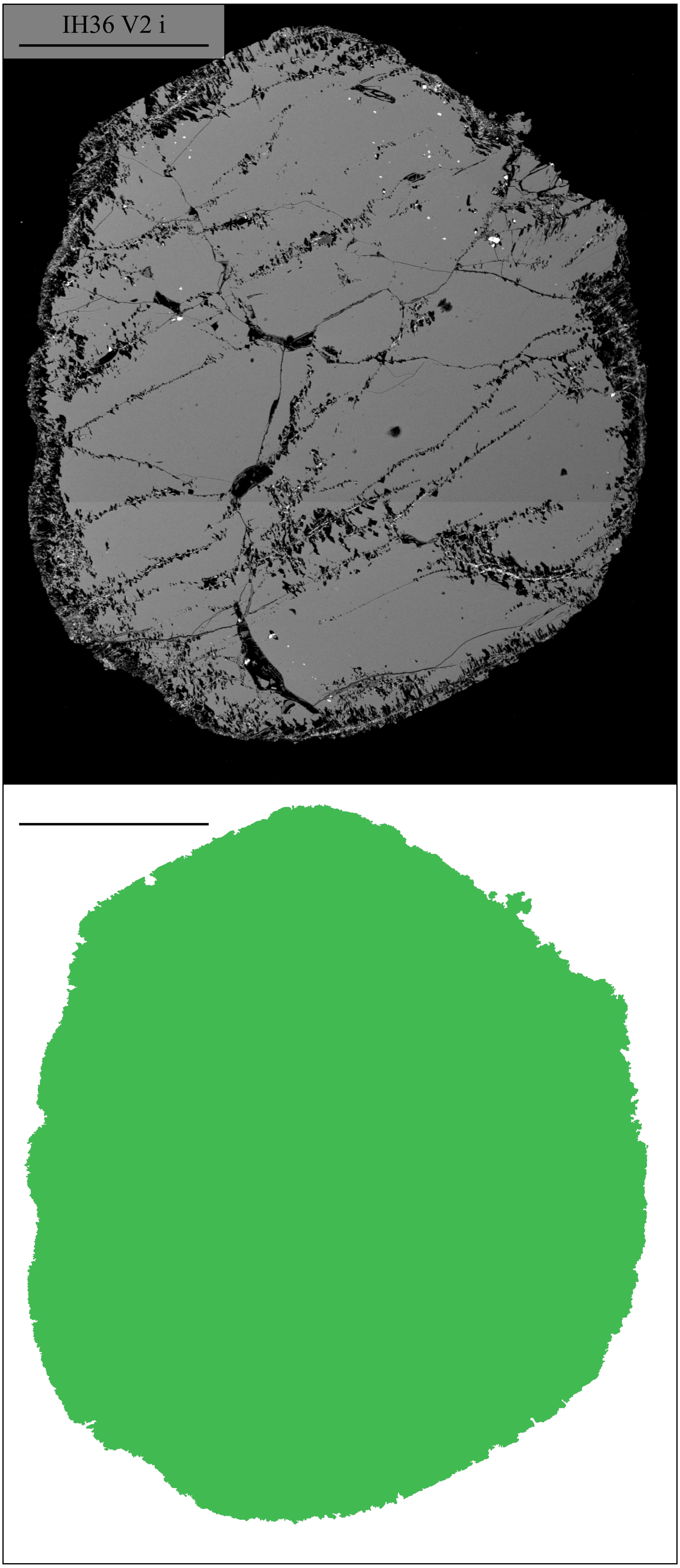
IH36 V2 i

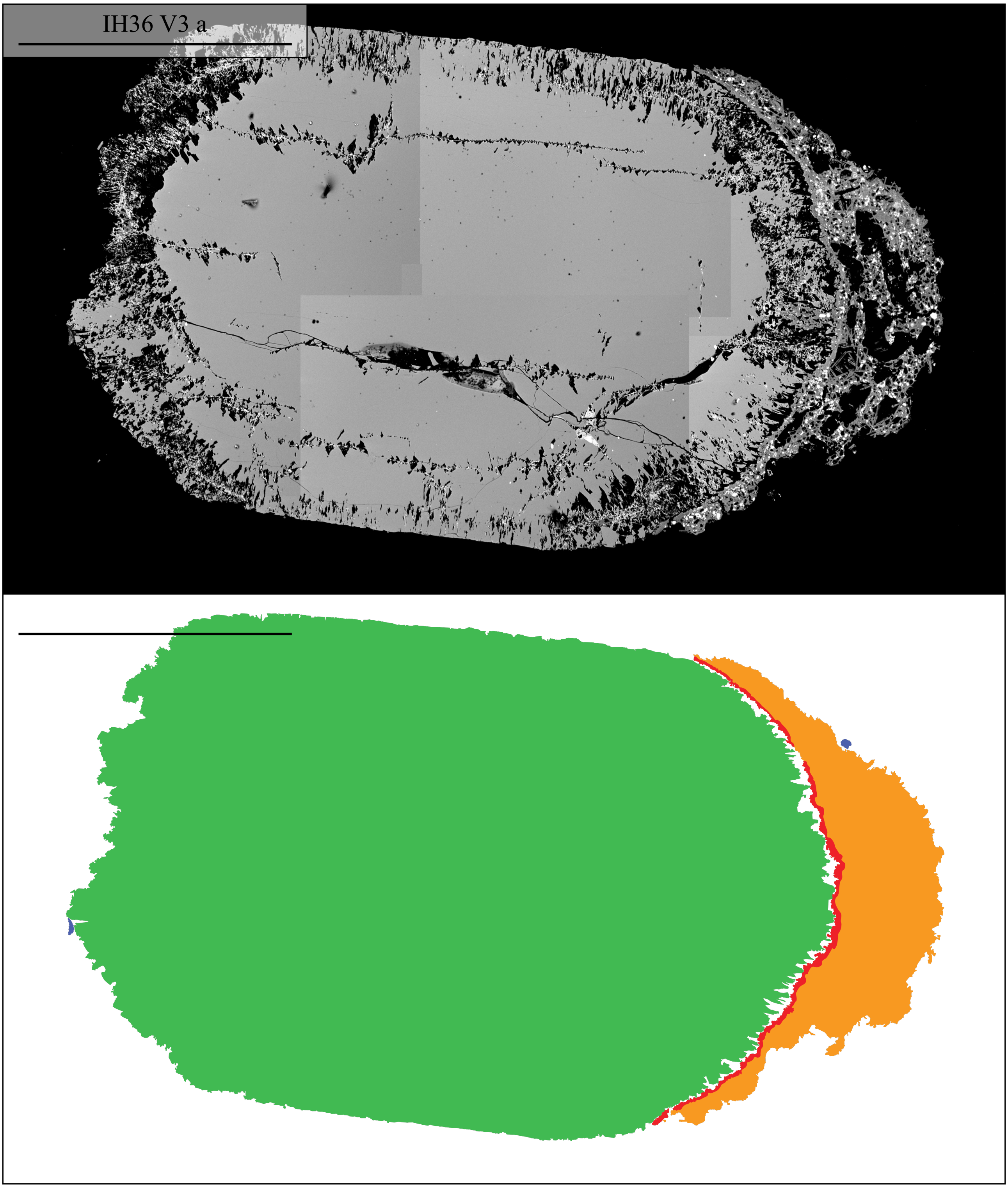
IH36 V3 a

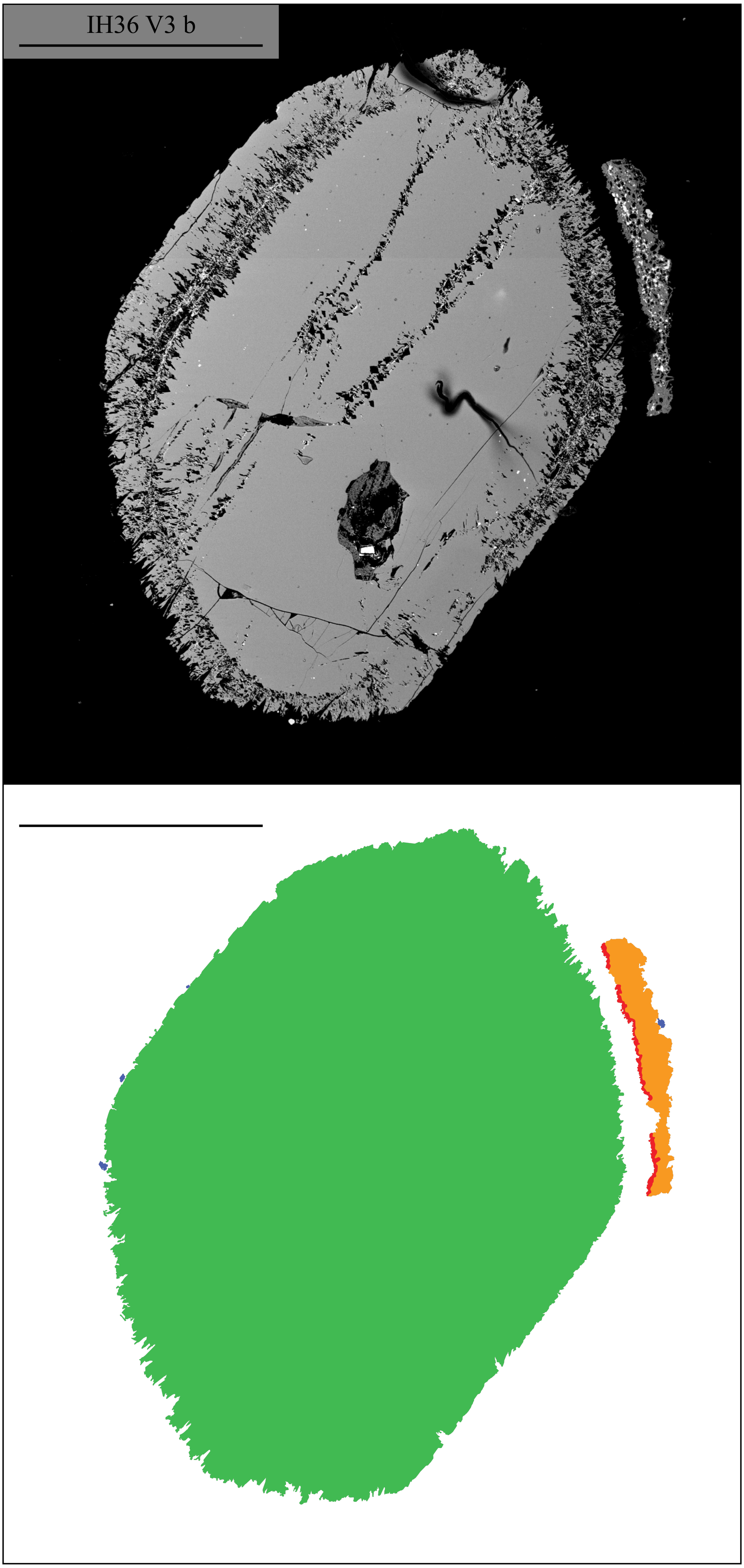
IH36 V3 b

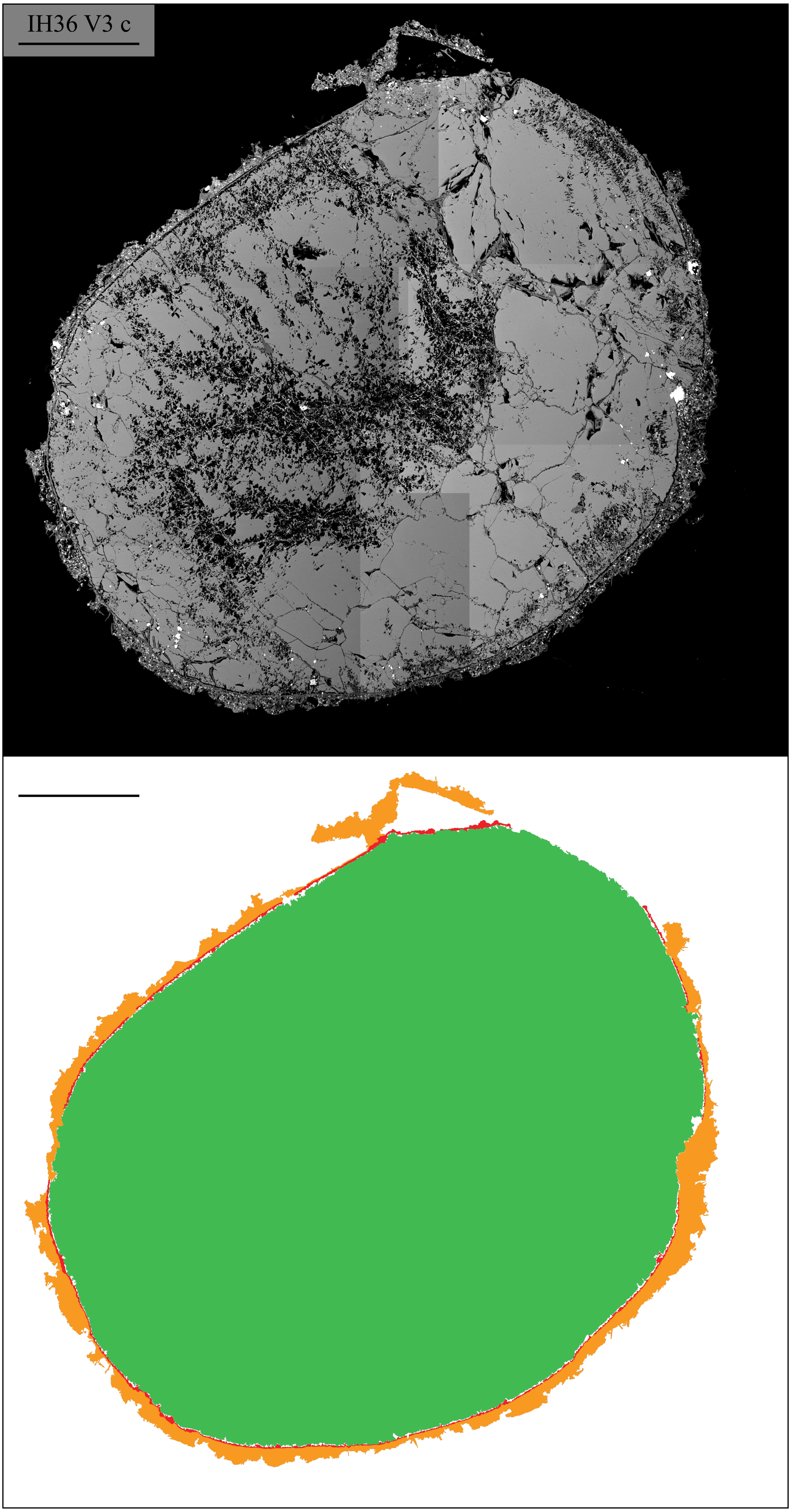
IH36 V3 c

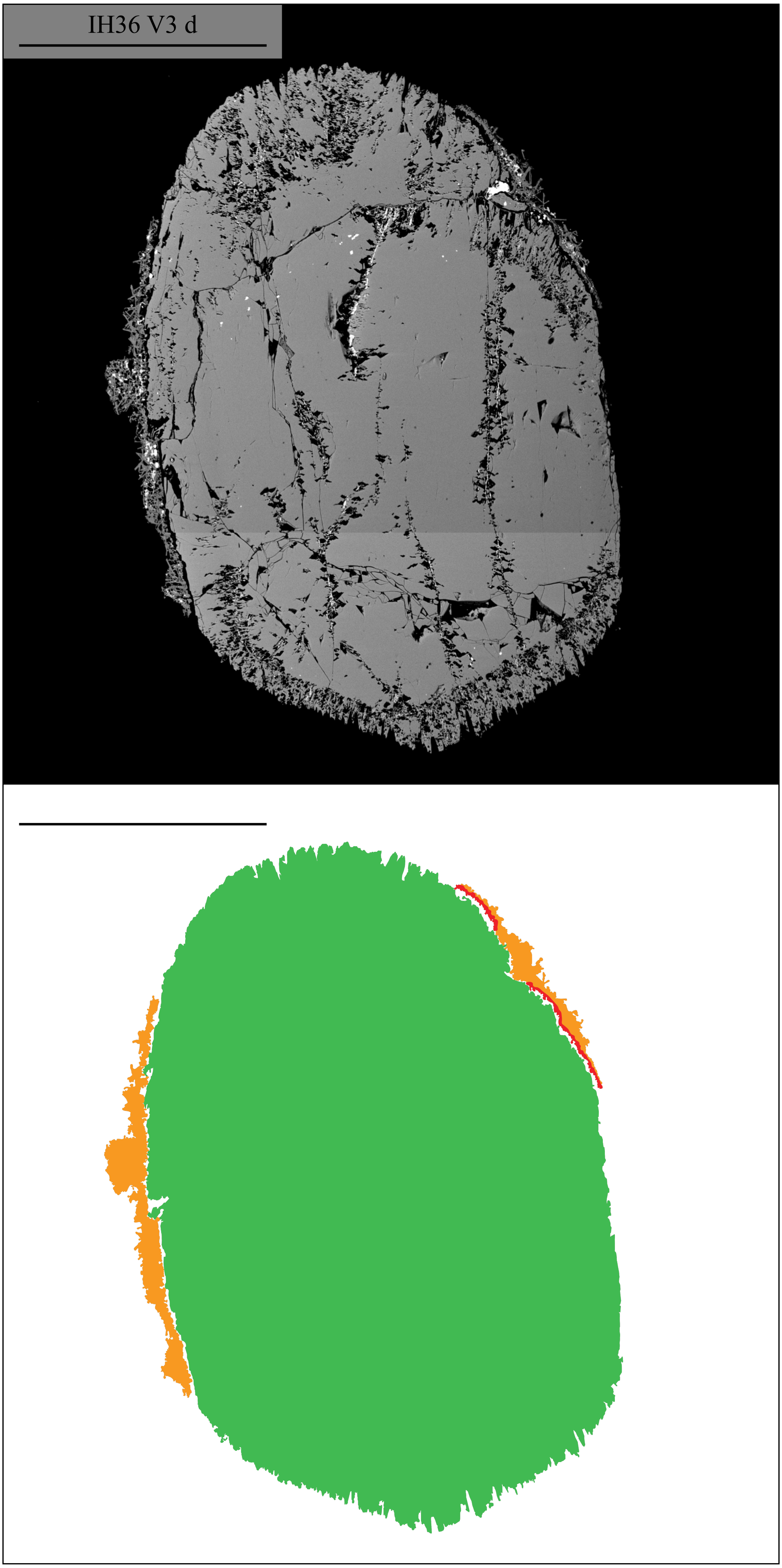
IH36 V3 d

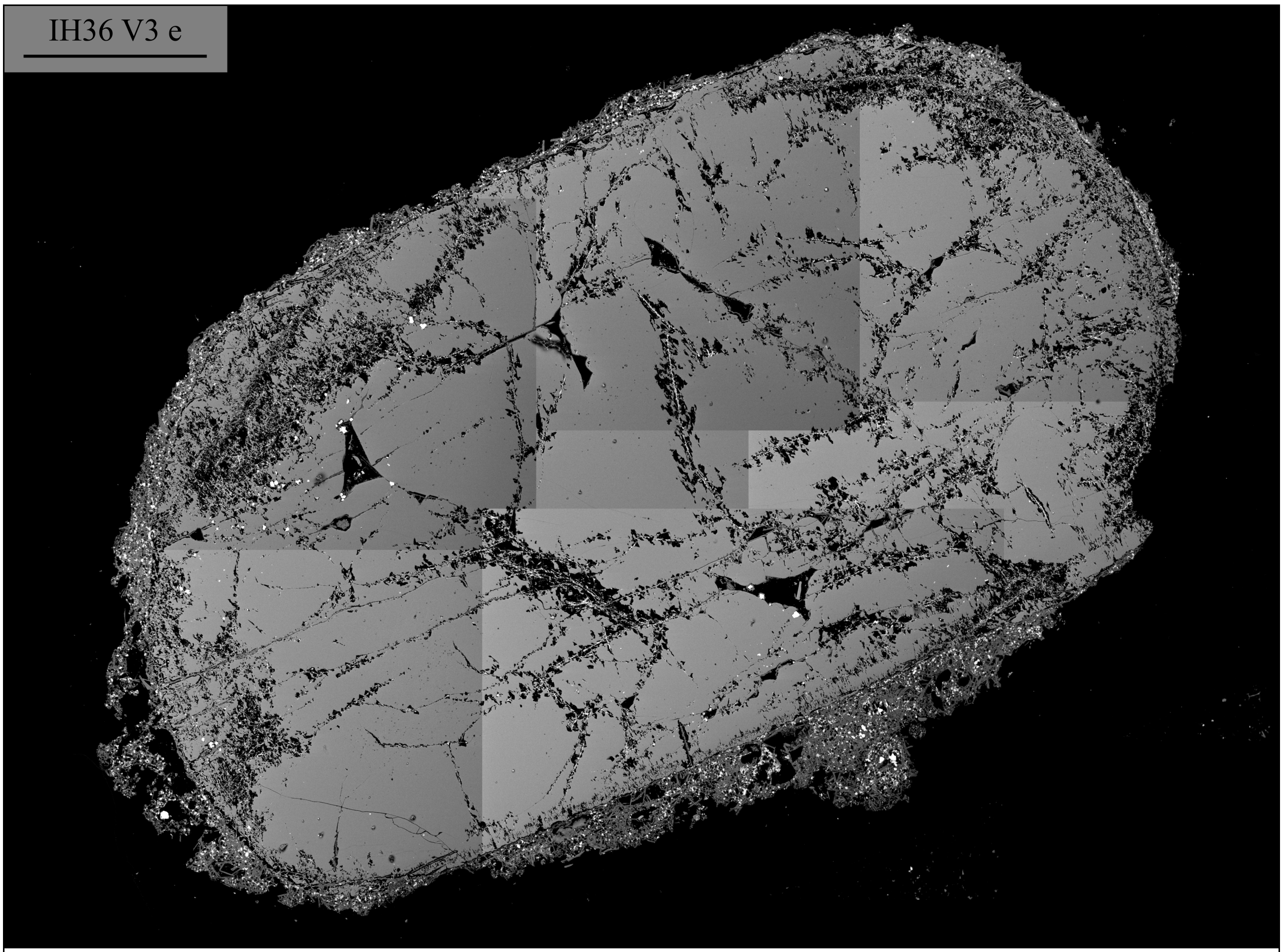
IH36 V3 e

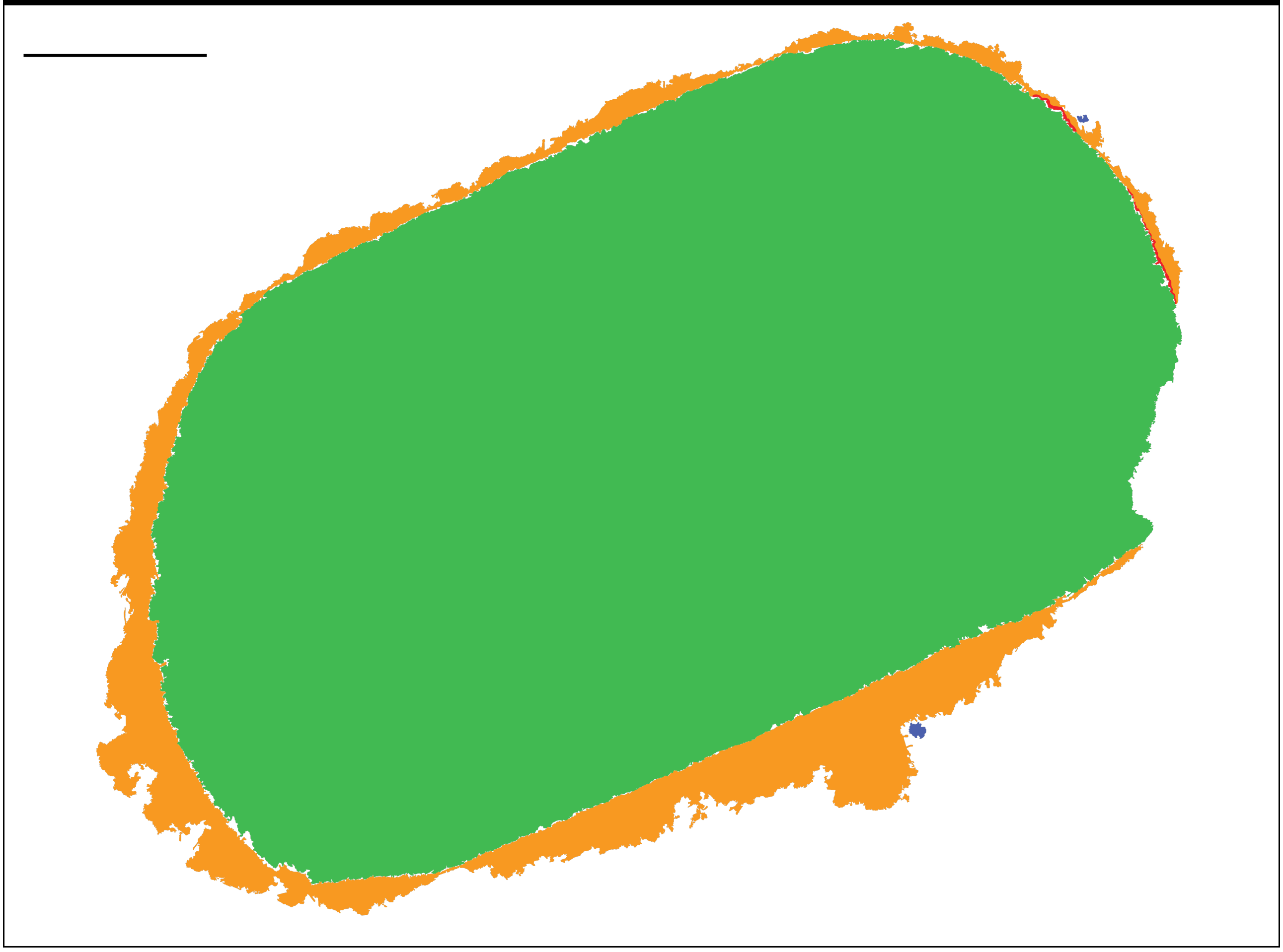

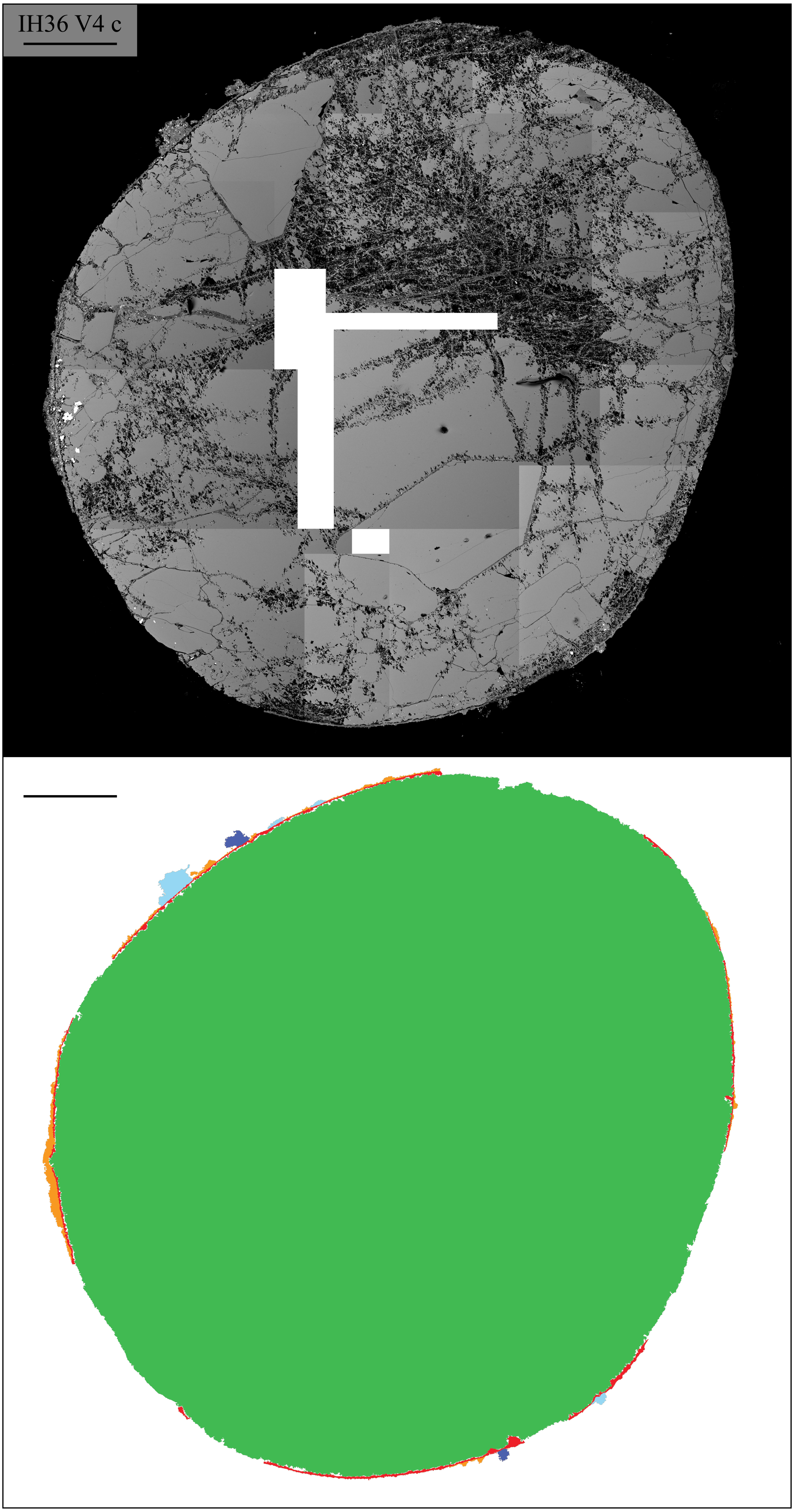
IH36 V4 c

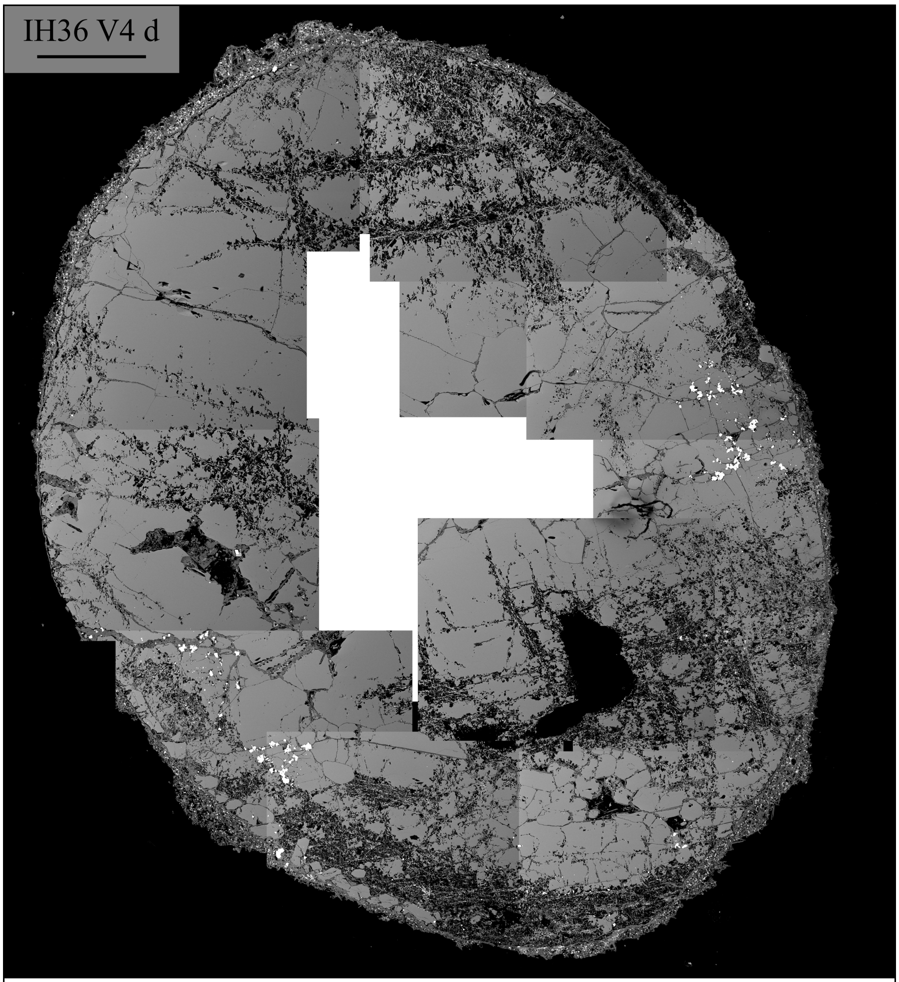
IH36 V4 d

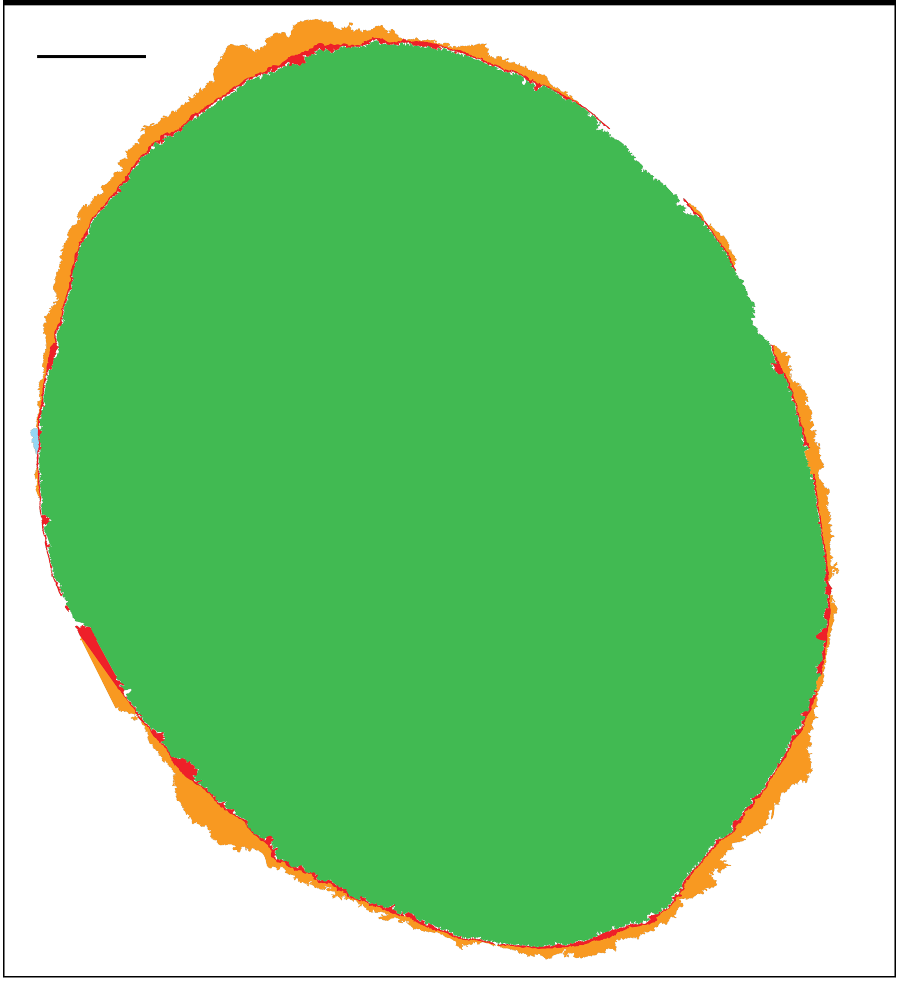

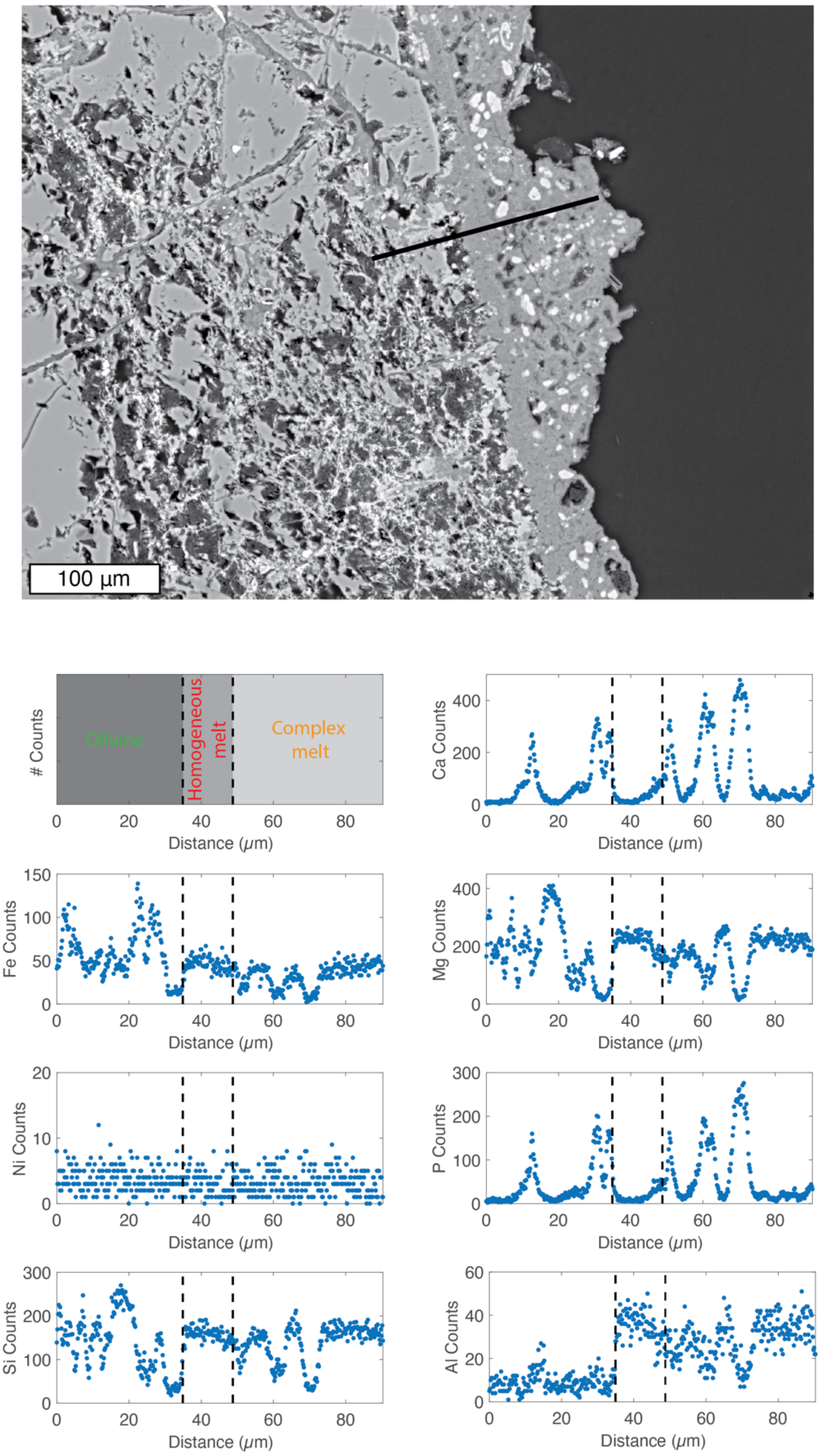

Fig. S2. Energy-dispersive X-ray spectroscopy (EDS) analysis from a line scan. The line position is marked on the SEM image and transects the olivine crystal, inner homogenous melt, and outer complex melt. These transitions from olivine to homogeneous melt and from homogeneous melt to complex melt are marked by black vertical dashed lines on the spectra. For all spectra, no chemical differences are observed between the homogeneous and complex melts (now glasses).
